# Non-invasive in vivo imaging of changes in Collagen III turnover in myocardial fibrosis

**DOI:** 10.1038/s44303-024-00037-z

**Published:** 2024-09-17

**Authors:** Nadia Chaher, Sara Lacerda, Giuseppe Digilio, Sergio Padovan, Ling Gao, Begoña Lavin, Rachele Stefania, Carlos Velasco, Gastão Cruz, Claudia Prieto, René M. Botnar, Alkystis Phinikaridou

**Affiliations:** 1 School of Biomedical Engineering and Imaging Sciences, King’s College London, 4th Floor, Lambeth Wing, St Thomas’ Hospital, London, SE17EH UK; 2 Centre de Biophysique Moléculaire, CNRS UPR 4301, Université d’Orléans rue Charles Sadron, 45071 Orléans, France; 3 Department of Science and Technological Innovation, Università del Piemonte Orientale, Alessandria, Italy; 4 Institute for Biostructures and Bioimages (CNR), Molecular Biotechnology Center, Torino, Italy; 5 Department of Biochemistry and Molecular Biology, School of Chemistry, Complutense University, Madrid, Spain; 6 Department of Radiology, University of Michigan, Ann Arbor, MI USA; 7 Escuela de Ingeniería, Pontificia Universidad Católica de Chile, Santiago, Chile; 8 King’s BHF Centre of Excellence, Cardiovascular Division, London, UK; 9 Instituto de Ingeniería Biológica y Médica, Pontificia Universidad Católica de Chile, Santiago, Chile

**Keywords:** Molecular imaging, Magnetic resonance imaging, Chemical biology

## Abstract

Heart failure (HF) affects 64 million people globally with enormous societal and healthcare costs. Myocardial fibrosis, characterised by changes in collagen content drives HF. Despite evidence that collagen type III (COL3) content changes during myocardial fibrosis, in vivo imaging of COL3 has not been achieved. Here, we discovered the first imaging probe that binds to COL3 with high affinity and specificity, by screening candidate peptide-based probes. Characterisation of the probe showed favourable magnetic and biodistribution properties. The probe’s potential for in vivo molecular cardiac magnetic resonance imaging was evaluated in a murine model of myocardial infarction. Using the new probe, we were able to map and quantify, previously undetectable, spatiotemporal changes in COL3 after myocardial infarction and monitor response to treatment. This innovative probe provides a promising tool to non-invasively study the unexplored roles of COL3 in cardiac fibrosis and other cardiovascular conditions marked by changes in COL3.

## Introduction

Ischaemic heart disease with myocardial infarction (MI) and ensuing heart failure (HF) is a leading cause of morbidity and mortality worldwide^1^. Collagen is the main structural protein of the cardiac extracellular matrix (ECM) constituting about 2–4% of the healthy adult human heart. Collagen type I (COL1) and collagen type 3 (COL3) represent about 85% and 11% of the total collagen content, respectively^2,3^. These two collagen types have significantly different mechanical properties. COL1 provides tensile strength and stiffness whereas COL3 provides elasticity^4^. Following MI, cardiac fibroblasts trans-differentiate into α-smooth muscle actin (α-SMA)–positive myofibroblasts that secrete collagens^5^. COL3 increases in the initial stages of cardiac remodelling and is later replaced by COL1^4^. Changes in the amount and ratio of COL1 to COL3 alter the biomechanics of the heart, impair cardiac function, and drive HF^6,7^.

In vivo detection of myocardial fibrosis remains challenging. Although circulating biomarkers relating to collagen turnover associate with myocardial fibrosis, their levels can be affected by other factors and thus are not cardiac-specific. This underscores the need to detect fibrosis directly in the heart^8–10^. To date, invasive biopsy still remains the gold-standard method to assess quantitative and qualitative changes in myocardial fibrosis^11,12^. Cardiac magnetic resonance (CMR) has emerged as the non-invasive imaging modality of choice to diagnose myocardial fibrosis safely, at high spatial resolution and without ionising radiation^11,13,14^. CMR using non-targeted gadolinium probes can detect replacement fibrosis as macroscopic regions with late gadolinium enhancement (LGE) and interstitial fibrosis by measuring an increase in the extracellular volume (ECV)^15–17^ using T_1_ mapping before and after gadolinium administration. An increase in native T_1_ driven by an increase in the interstitial space, such as that observed after infarction, has also been used for diagnosing fibrosis^13^. Despite their clinical value, LGE imaging, native T_1_ mapping and changes in ECV using non-targeted gadolinium agents can also be caused by other factors (e.g. oedema^13^, lipid overload^18^) and thus may not be specific to fibrosis. Importantly, CMR does not directly measure fibrosis and cannot differentiate between collagen subtypes. Therefore, development and optimisation of molecular probes for specific imaging of collagen types are needed to detect fibrosis and monitor therapeutic response.

Molecular magnetic resonance imaging (MRI) agents that target active fibrogenesis (e.g., reactive aldehydes), and ECM proteins such as COL1, COL5, elastin and tropoelastin^19–23^ have enabled imaging of tissue fibrosis at the molecular level in experimental models^19,21,22,24–33^. Of these, probes targeting COL1 and elastin enabled superior detection of myocardial fibrosis compared with non-targeted probes used in the clinic^19,34–36^. Additionally, radiolabelled fibroblast activation protein inhibitors (FAPI) have been used for nuclear molecular imaging of activated fibroblasts as a strategy to image fibrosis^37–40^. Despite COL3 being one of the major contributors to myocardial fibrosis after MI, the lack of an imaging probe that targets COL3 has halted our ability to probe the dynamic spatiotemporal changes in COL3 remodelling and understand the diagnostic, therapeutic and functional roles of COL3 in post-MI cardiac remodelling. Here, we developed the first COL3-binding probe suitable for molecular MRI. Using this probe, we detect for the first-time changes in the natural turnover of COL3 non-invasively in a murine model of MI. We also show that the newly developed COL3 probe can detect changes in COL3 turnover following MI after treatment with the angiotensin-converting enzyme (ACE) inhibitor, enalapril.

## Results

### Molecular probes

**Fig. 1:**
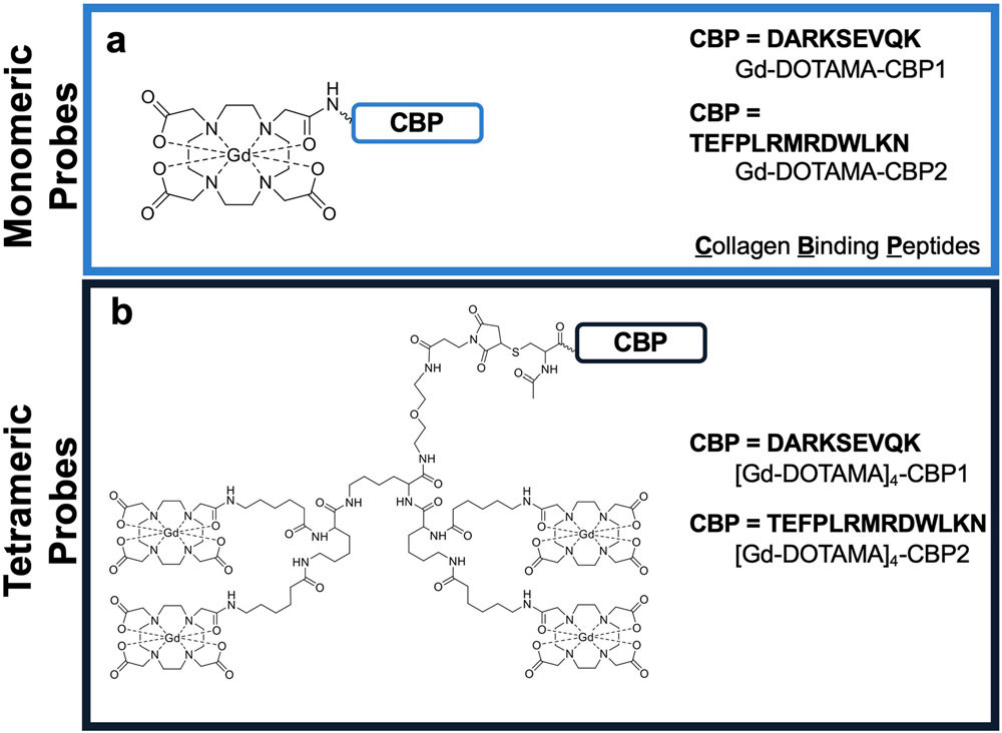
Chemical structures of the MRI probes based on COL3 binding peptides. **a** Monomeric probes and **b** Tetrameric probes.

Two peptides were identified as potential COL3 binders, DARKSEVQK (Collagen Binding Peptide 1, CBP1) and TEFPLRMRDWLKN (CBP2)^41–43^. These peptides were conjugated at the N-terminus with either one or four Gd-DOTA-like complexes to obtain monomeric or tetrameric molecular probes, respectively (Fig. 1). The monomeric probes were obtained by linking one DOTA chelator to the N-terminus amino group of the collagen-binding peptide, yielding DOTA-monoamide collagen conjugates (DOTAMA-CBPs). Then, complexation with Gd(III) yielded the probes for molecular imaging by MRI (compounds Gd-DOTAMA-CBP1 and Gd-DOTAMA-CBP2). Complexation of the very same DOTAMA-CBPs with Eu(III) or with ^68^Ga(III) yielded molecular probes for Eu(III) Time Resolved Fluorescence (TRF) binding assays or tracers for Positron Emission Tomography (PET) pharmacokinetic and biodistribution studies, respectively. To increase the payload of contrast agents per single binding event, we also developed tetrameric molecular probes carrying four Gd-DOTA-like units per single molecule. In such tetrameric probes, a tri-lysine scaffold bearing four Gd-DOTAMA units was conjugated at the N-terminus of collagen binding peptides through a flexible spacer to minimise potential steric hindrance to COL3 binding (compounds [Gd-DOTAMA]_4_-CBP1 and [Gd-DOTAMA]_4_-CBP2) (Supplementary Fig. 1a, b).

Scrambled versions (same amino acids but in a randomised arrangement) of both the monomeric and tetrameric probes were also synthesised. The sequences RDKKVAEQS and EWFMKDRLLNRPT were used to obtain scrambled CBP1 (SccCBP1) and scrambled CBP2 (SccCBP2), respectively, to be used as negative controls.

### In vitro binding assays

**Fig. 2:**
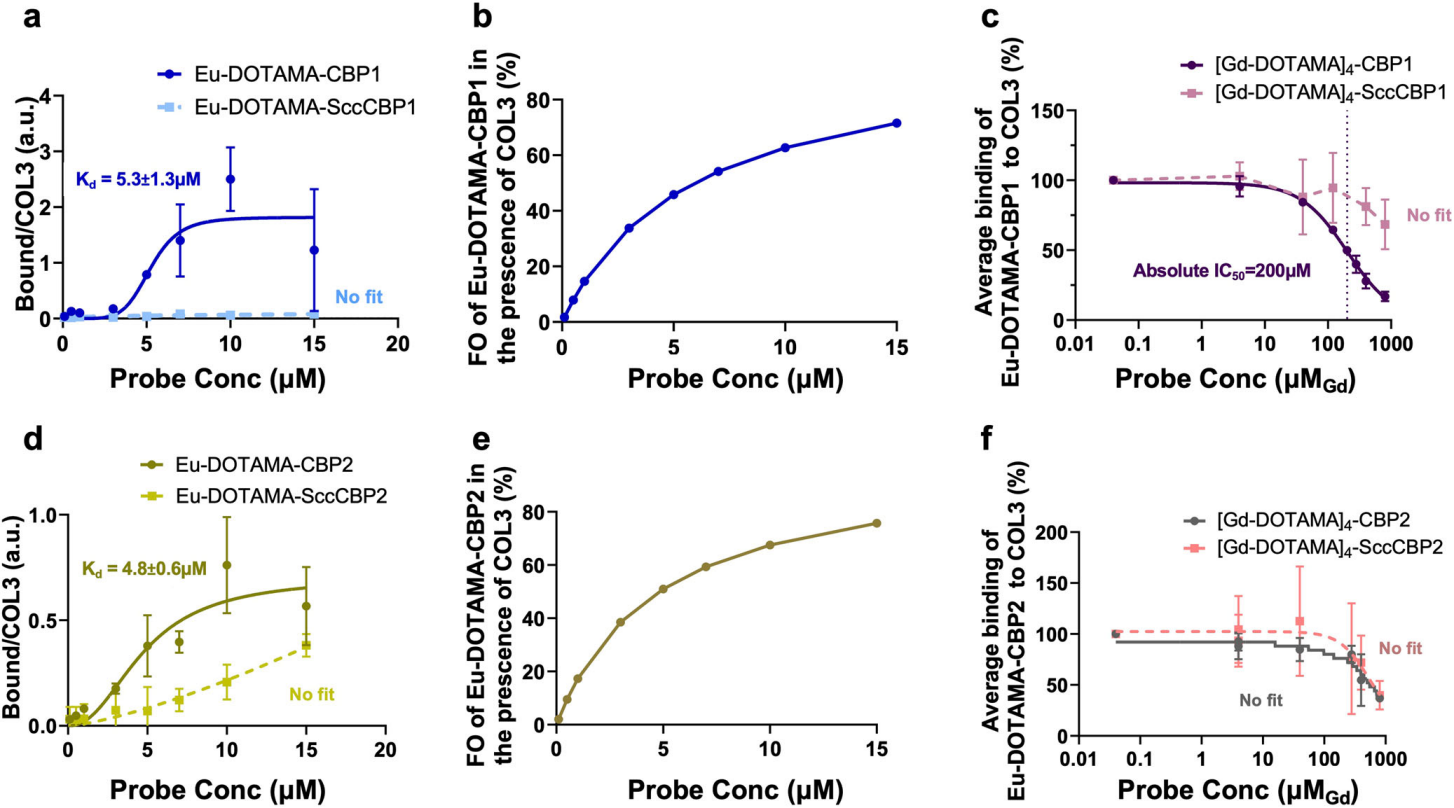
In vitro binding assays to assess the binding affinity and specificity of the COL3 probes. **a** The Eu-DOTAMA-CBP1 probe binds to COL3 with good affinity and specificity. **b** The FO of the Eu-DOTAMA-CBP1 in the presence of COL3 is 74%. **c** Competition assay between monomeric Eu-DOTAMA-CBP1 and tetrameric [Gd-DOTAMA]_4_-CBP1 shows that the tetramer can displace the monomer from COL3 binding sites. **d** The Eu-DOTAMA-CBP2 probe binds to COL3 but with a significant non-specific contribution. **e** The FO of the Eu-DOTAMA-CBP2 in the presence of COL3 is 76% but binding is unspecific. **f** Competition assay between monomeric Eu-DOTAMA-CBP2 and tetrameric [Gd-DOTAMA]_4_-CBP2 shows a small difference between the displacement curves of [Gd-DOTAMA]_4_-CBP2 and its scrambled counterpart, suggesting that the binding is largely unspecific. CBP collagen binding peptide, SccCBP scrambled version of the collagen binding peptide, Eu europium, Gd gadolinium, FO fractional occupancy.

The binding isotherms of the monomeric probes Eu-DOTAMA-CBP1 and Eu-DOTAMA-CBP2 towards COL3 obtained by the Eu(III) TRF plate assay as we previously described^21,31,44^ are shown in Fig. 2. Eu-DOTAMA-CBP1 bound to COL3 with a good affinity (*K*_*d*_ = 5.3 ± 1.3 µM), (Fig. 2a), and a fractional occupancy (FO) at the highest concentration of probe of 74% (Fig. 2b). Importantly, the scrambled Eu-DOTAMA-SccCBP1 probe used as the negative control showed no binding to COL3, confirming the specificity of the binding interaction between Eu-DOTAMA-CBP1 and COL3 (Fig. 2a). By contrast, there was little binding of the CBP1 probe to COL1, and further control experiments with the Scc-CBP1 probe indicated that such binding was non-specific (Supplementary Fig. 2a). Similarly, the probe did not bind to both elastin and albumin, further verifying its specificity towards COL3 (Supplementary Fig. 2b, c).

The Eu-DOTAMA-CBP2 probe apparently bound to COL3 with a similar binding affinity (*K*_*d*_ = 4.8 ± 0.6 µM) and fractional occupancy (76%) as the Eu-DOTAMA-CBP1 probe (Fig. 2d, e). However, the scrambled probe (negative control) also exhibited binding to COL3, thus indicating a significant lower specific binding of CBP2 towards COL3. The Eu-DOTAMA-CBP2 probe also showed some binding to COL1, but control experiments using the scrambled probe indicated that such binding was non-specific. No binding was detected towards elastin and albumin (Supplementary Fig. 2d–f).

The binding affinity of the tetrameric probes to COL3 was studied by competition experiments, in which the Gd(III)-labelled tetrameric probe was used to displace the corresponding Eu(III)-labelled monomeric counterpart from COL3 binding sites, as shown in Fig. 2c, f. Importantly, the plot shown in Fig. 2c shows that the Gd-[DOTAMA]_4_-CBP1 probe can displace its monomeric Eu(III)-labelled counterpart from COL3 binding sites (with IC_50_ = 200 μM), while the scrambled Gd-[DOTAMA]_4_-SccCBP1 probe cannot. This finding clearly indicates that both the tetrameric [Gd-DOTAMA]_4_-CBP1 probe and its monomeric Eu-DOTAMA-CBP1 counterpart do specifically bind to COL3. Crucially, no displacement would have been observed if the binding of either one of the two ligands (i.e. the monomeric or the tetrameric probe) were non-specific.

A different result was found for the CBP2-based probes (Fig. 2f). Here, the displacement of the monomeric Eu-DOTAMA-CBP2 can be observed only at very high concentrations of competing [Gd-DOTAMA]_4_-CBP2 and, most importantly, there is no difference between the displacement curves of [Gd-DOTAMA]_4_-CBP2 and its scrambled counterpart (Fig. 2f). This indicates that the CBP2-based probes exhibit significant non-specific binding in these assay conditions.

### Relaxivity studies

To further characterise the molecular characteristics of these probes, the ^1^H nuclear magnetic relaxation dispersion profiles (^1^H-NMRD) of Gd-DOTAMA-CBP1, Gd-DOTAMA-CBP2 and [Gd-DOTAMA]_4_-CBP1 were acquired in phosphate buffer saline (PBS) and in 0.6 mM human serum albumin (HSA), at 25 and 37 °C (Supplementary Fig. 3a–c). The profiles obtained were characteristic of small-molecular weight (MW) complexes, even when in the presence of HSA. Table 1 summarises the relaxivity values at 20 MHz and 37 °C, for all probes and for Gadovist^45^ a nonpeptide-based (non-targeted) clinically approved contrast agent^39^. As anticipated, the new probes demonstrated higher relaxivity with *r*_1_ values of 7.4 and 8.8 mM^−1^s^−1^ for Gd-DOTAMA-CBP1 and Gd-DOTAMA-CBP2, respectively. These values were about 2-fold higher than the clinically approved agent (Gadovist; 3.7 mM^−1^s^−1^) and were within the range of other small MW probes based on Gd-DOTAMA, such as TESMA (11.7 mM^−1^s^−1^)^19,21^. The increased relaxivity can be attributed to the higher MW of the targeted probes resulting in increased rotational correlation time and to the presence of water molecules around the peptide-targeting moiety contributing to second- and outer- sphere effects. The tetrameric probe showed a small decrease in the relaxivity per Gd, due to the high flexibility of the linker, irrespective of its higher MW as previously observed for other peptide-tetrameric probes^46^.

**Table 1:** Relaxivities of monomeric and tetrameric probes bearing CBP1 and CBP2 peptide moieties, in PBS and 0.6 mM human serum albumin (HAS) at 20 MHz and 37 °C

Compound^$^	*r*_1_ mM (mM^−1^ s^−1^)	
	PBS	HSA 0.6 mM
Gadovist^45^	3.7	6.1*
Gd-DOTAMA-CBP1	7.4 ± 0.1	8.3 ± 0.2
Gd-DOTAMA-CBP2	8.8 ± 0.2	10.4 ± 0.2
[Gd-DOTAMA]_4_-CBP1		
• per molecule	27.2 ± 0.4	28.6 ± 0.4
• per Gd(III)	6.8 ± 0.1	7.2 ± 0.1

^$^ All Gd concentrations were confirmed by inductively coupled plasma (ICP) and/or bulk magnetic susceptibility (BMS)^90^; * in non-sterile bovine plasma (corresponding to an albumin concentration of 0.64 to 0.82 mmol/L)^19,21,45,50^.

### In vivo PET/CT biodistribution experiments

**Fig. 3:**
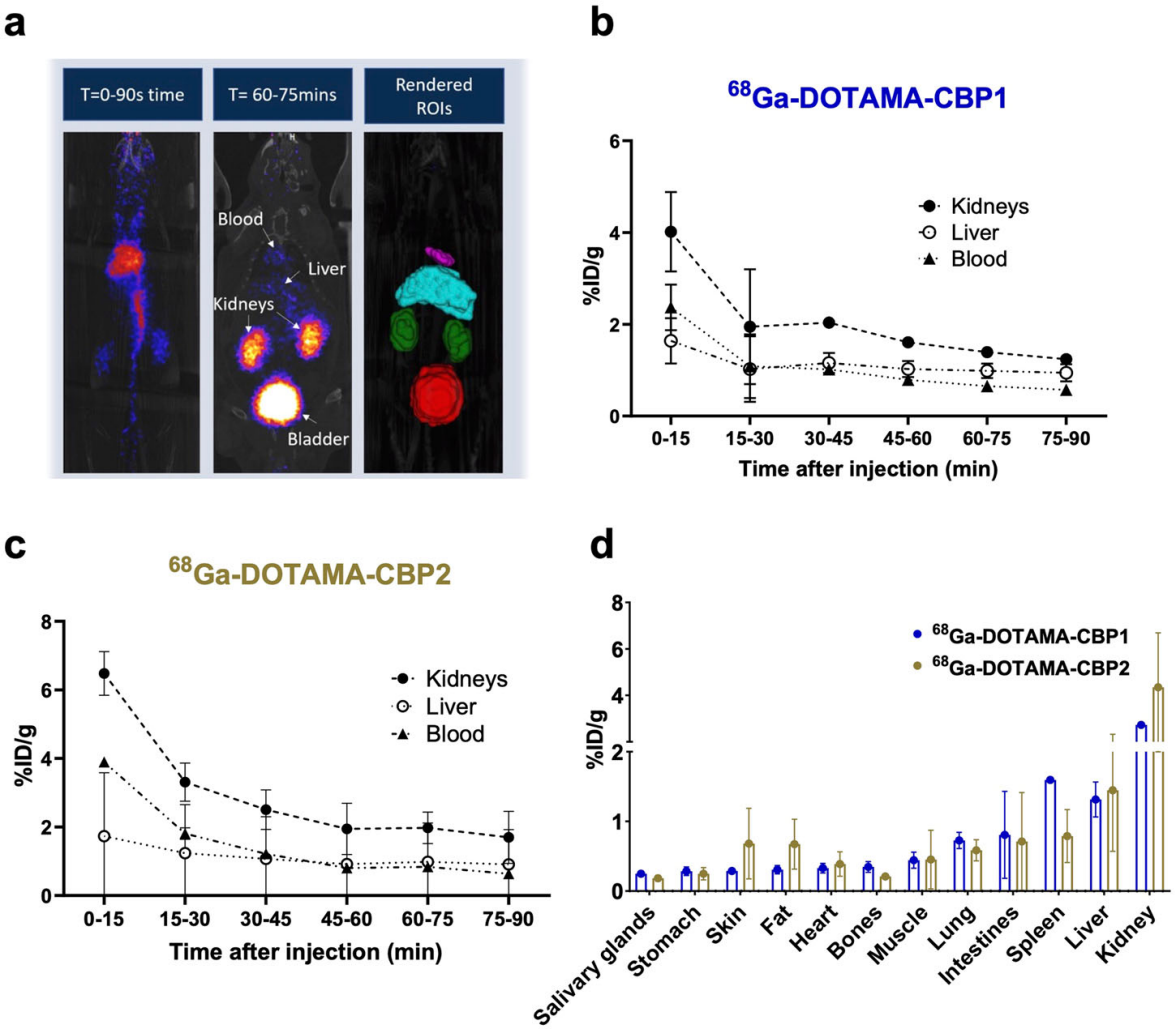
In vivo PET/CT pharmacokinetics and biodistribution using ^68^Gallium labelled probes. **a** Fused PET and CT images demonstrate the clearance pathway and segmentation of the organs for quantitative analysis. **b**, **c** Quantification of injected dose of the ^68^Ga-DOTAMA-CBP1 (**b**) and the ^68^Ga-DOTAMA-CBP2 (**c**) probes in the kidney, liver, and blood over time shows favourable pharmacokinetics. **d** Ex vivo radioactivity measurements show high kidney uptake and low uptake in all other tissues. T time post-injection (minutes).

In vivo biodistribution experiments of the ^68^Ga-DOTAMA-CBP1 and ^68^Ga-DOTAMA-CBP2 probes in mice showed fast blood clearance, renal excretion, and no significant unspecific uptake in tissues (Fig. 3a). Quantification of the injected dose showed a fast first phase clearance from the blood with most of the probe eliminated within 15–30 minutes and a slower elimination phase between 30–90 min (Fig. 3b,c). Ex vivo gamma counting biodistribution experiments used to precisely quantify radioactivity in tissues confirmed the in vivo observations (Fig. 3d), indicating that both probes are excreted by renal clearance. These findings highlight the probes’ potential for targeted imaging and in vivo applications without unspecific retention in tissues and a safe elimination profile.

### Identification of the lead probe using a murine model of vascular remodelling

**Fig. 4:**
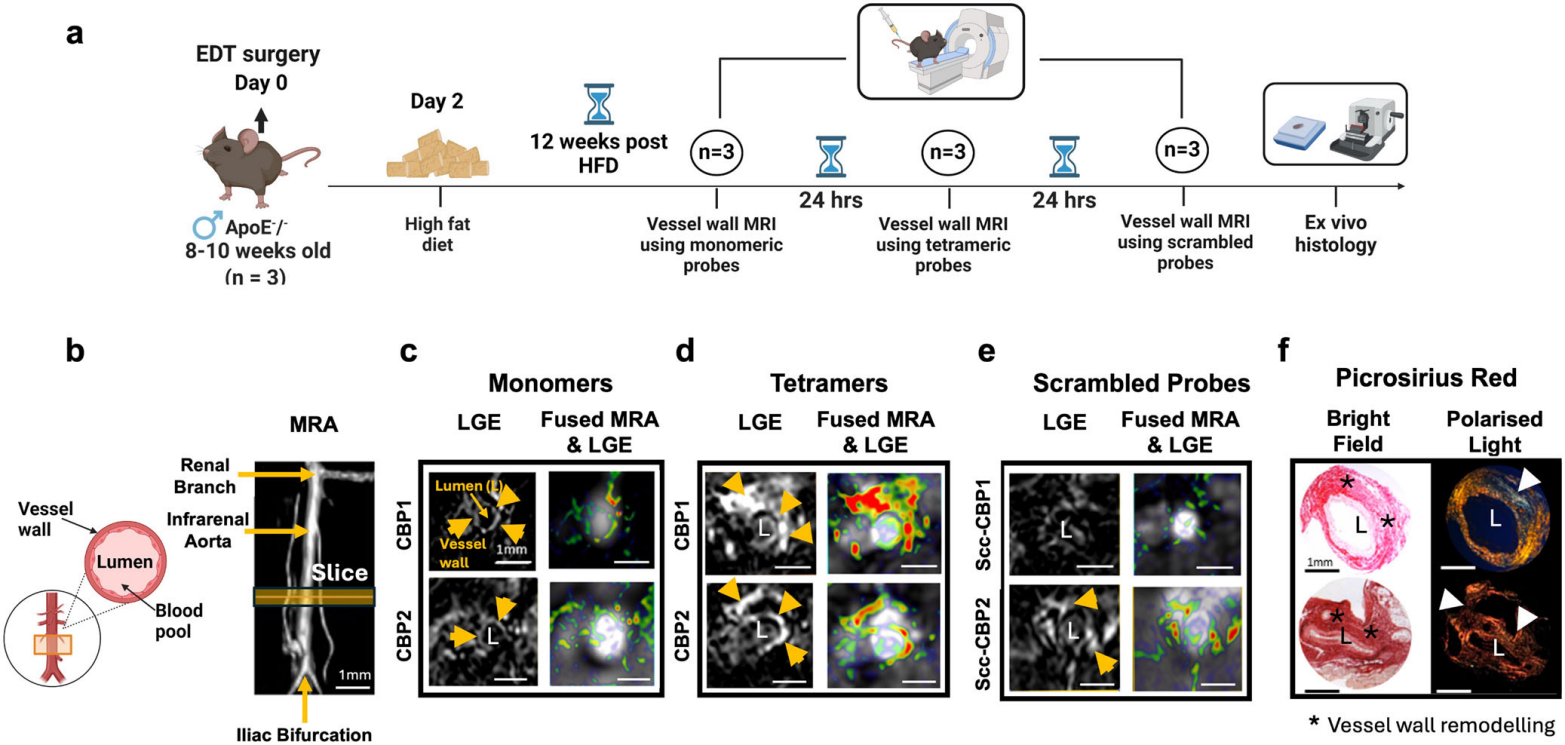
In vivo MRI comparison of the monomeric and tetrameric probes in a murine model of vascular remodelling. **a** Experimental design. **b** Schematic of the infrarenal aorta undergoing surgery and magnetic resonance angiography (MRA) segment. **c** Both monomeric probes show enhancement up to 60 min post- injection. **d** [Gd-DOTAMA]_4_-CBP1 and [Gd-DOTAMA]_4_-CBP2 show stronger enhancement at 60 min compared with corresponding monomeric probes **e** The scrambled tetrameric Scc-CBP1 probe shows negligible enhancement but the Scc-CBP2 probe shows enhancement suggesting unspecific binding of the [Gd-DOTAMA]_4_-CBP2 probe. **f** Signal enhancement observed by MRI co-localised with COL3 fibres (green) as seen by histology. L aortic lumen, HFD high-fat diet. Figure 4a and schematic in 4b were created with BioRender.com.

To select the lead probe for cardiac CMR experiments, we first directly compared the MR imaging properties of the monomeric and tetrameric Gd(III)-labelled probes to detect COL3 remodelling in a murine model of aortic injury resulting in collagen remodelling^47^. This strategy was chosen because for multiple probe comparisons involving repeated scans imaging the vessel wall is technically more practical than the heart. After aortic injury and 12 weeks of high-fat feeding, each mouse was imaged up to 2 h after injection of the monomeric probe (24 h later) using the tetrameric probe (another 24 h later) with the scrambled probe. Finally, tissues were excised for histology (Fig. 4a). Magnetic Resonance Angiography (MRA) and late gadolinium enhancement (LGE) images are shown in Figs. 4b–e. We observed signal enhancement within the remodelled aortic wall using both the monomeric and the tetrameric probes carrying the CBP1. There was enhancement from the monomeric probe with the best imaging acquisition at 60 min post-injection. In comparison, the tetrameric probe showed stronger and persistent signal for up to 120 min post-injection (Supplementary Fig. 4a), prolonging the imaging acquisition window compared with its monomeric counterpart. No signal enhancement was observed using the scrambled [Gd-DOTAMA]_4_-SccCBP1 probe, supporting the specificity of the CBP1-based probe towards COL3 in vivo. These in vivo results agree with the findings of the in vitro binding assays showing high affinity and specificity of the Eu-DOTAMA-CBP1 towards COL3. The probes carrying the CBP2, also showed enhancement of the remodelled aortic wall using the monomeric probe with the best imaging time at 60 min (Figs. 4b–e) and longer enhancement using the tetrameric CBP2 probe (Supplementary Fig. 4a). However, the scrambled [Gd-DOTAMA]_4_-SccCBP2 probe also showed signal enhancement, suggesting that the CBP2 is less-specific towards the target. These in vivo results also support the in vitro binding assays showing that the binding of the CBP2-based probes to COL3 is unspecific. We then stained sections with Picrosirius red and visualised them under polarised light (Fig. 4f). COL1 fibres are thick, strongly birefringent and appear yellow/ red whereas COL3 fibres are thinner, weakly birefringent and appear as green^48^. Collagen deposition within the remodelled aortic wall was increased containing a mixture of COL1 (yellow/red) and COL3 (green) fibres. Signal enhancement seen on the MRI coincided with COL3 fibres detected by histology (Fig. 4f). The specificity of binding was further confirmed using a fluorescent rhodamine-labelled CBP1 analogue probe. We observed co-localisation of the fluorescent probe with COL3-rich fibres within the remodelled aortic wall and no co-localisation to COL1 fibres (Supplementary Fig. 4b).

### Molecular imaging of COL3 after myocardial infarction

**Fig. 5:**
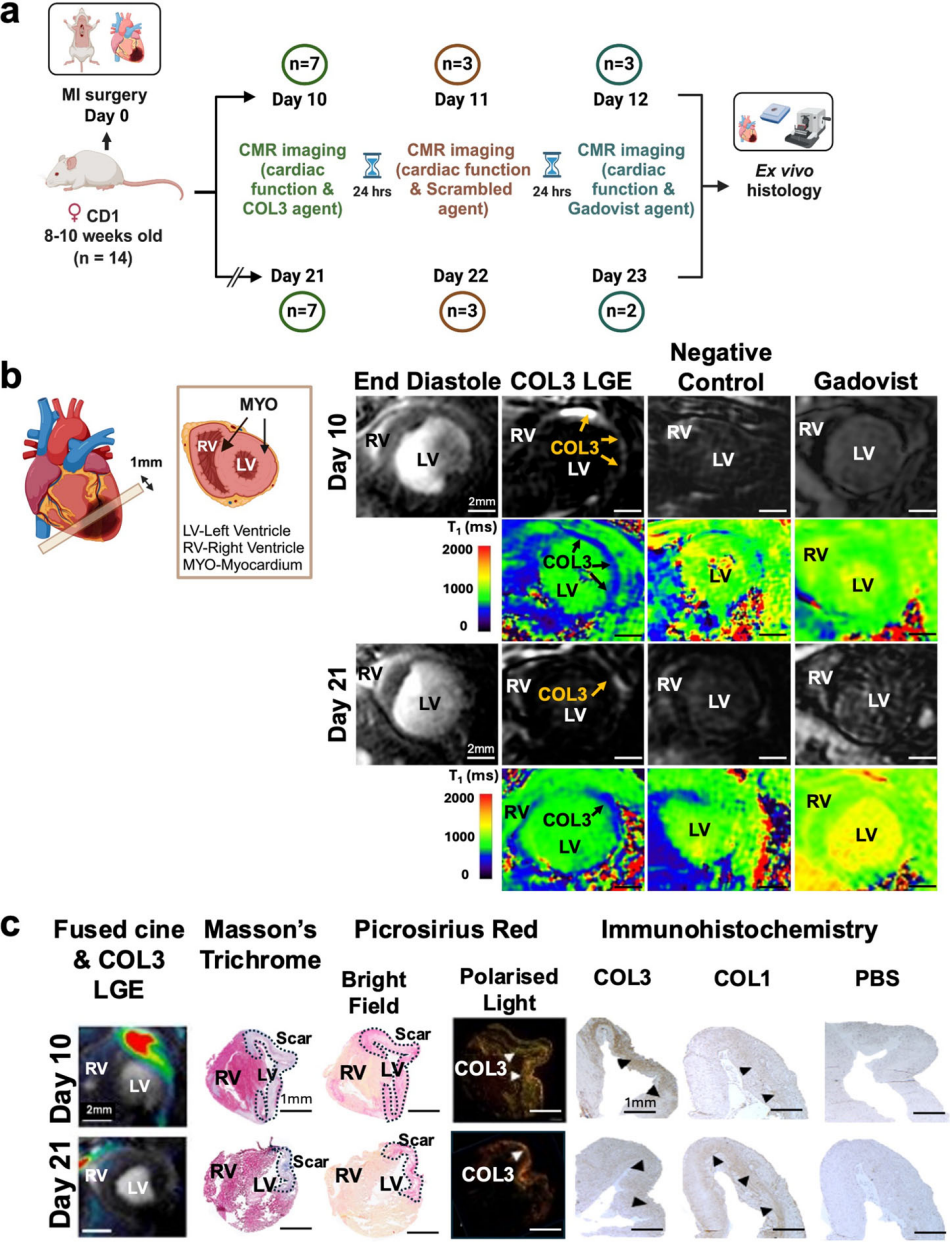
First in vivo molecular imaging of COL3 after myocardial infarction using the tetrameric [Gd-DOTAMA]_4_-CBP1 probe. **a** Experimental design. **b** Molecular CMR (at 60 min post-injection of the COL3 probe) shows strong signal enhancement and lower T_1_ values in the infarcted myocardium at day 10. Conversely, low signal enhancement and higher T_1_ values are observed in the infarct at day 21. No enhancement is observed with both the negative control probe and the non-targeted clinical probe (Gadovist) (at 30 minutes post-injection). **c** MRI signal enhancement co-localised with collagen seen on Masson’s trichrome and PSR staining in BF and with COL3 fibres (green fibres) seen with PLM. Immunohistochemistry validates co-localisation of the MRI signal with COL3 at day 10 and the drop in MRI signal at day 21 when COL3 is replaced by COL1. RV right ventricle, LV left ventricle, PSR Picrosirius Red, BF bright field, PLM polarised light microscopy. Figure 5a and schematic in 5b were created with BioRender.com.

Considering the results of the in vitro binding assays, the biodistribution and the MRI studies, the [Gd-DOTAMA]_4_-CBP1 probe emerged as the lead imaging probe for molecular imaging of COL3 post-MI induced by permanent occlusion of the proximal left anterior descending (LAD) coronary artery (Fig. 5a) Prior to imaging, we characterised the changes in the expression of COL3 post-MI by histology. These studies showed that COL3 increases 10 days post-MI and decreases by day 21 (Supplementary Fig. 5a). Guided by these results, we then used a clinical 3 Tesla MR scanner for molecular imaging of COL3 post-MI (Supplementary Fig. 5b). To establish the optimal time for imaging after injection of the probe, serial inversion recovery images of the left ventricle were obtained at 30, 60 and 90 min after injection of the probe at day 10 post-MI (Supplementary Fig. 5c). We observed signal enhancement in the infarcted myocardium at 30 min post-injection peaking at 60 min and significantly decreasing by 90 minutes. Based on these findings, subsequent imaging experiments were carried out at 60 min post-injection of the COL3 probe.

Molecular CMR enabled selective profiling of the natural turnover of COL3 post-MI (Fig. 5b). A strong signal enhancement was observed in the infarct 10 days post-MI, when COL3 is elevated, that decreased at day 21 when COL3 is replaced by COL1 (Fig. 5b). T_1_ maps showed higher probe uptake in the infarct (lower T_1_ values; blue colour) and significantly lower probe uptake in the remote myocardium (higher T_1_ values; green colour). Importantly, no enhancement was observed using the scrambled probe or the clinical agent Gadovist at both timepoints after MI. The imaging data were validated by histology showing co-localisation of the MRI signal with the scar visualised by trichrome and Picrosirius red straining (Fig. 5c). Importantly, immunohistochemistry validated the MRI observations showing a pronounced upregulation of COL3 expression (brown) within the infarct at day 10 and a decrease of COL3 by day 21. Conversely, negligible amount of COL1 was present at day 10 but COL1 increased by day 21.

### Molecular imaging of COL3 detects changes after treatment with enalapril

**Fig. 6:**
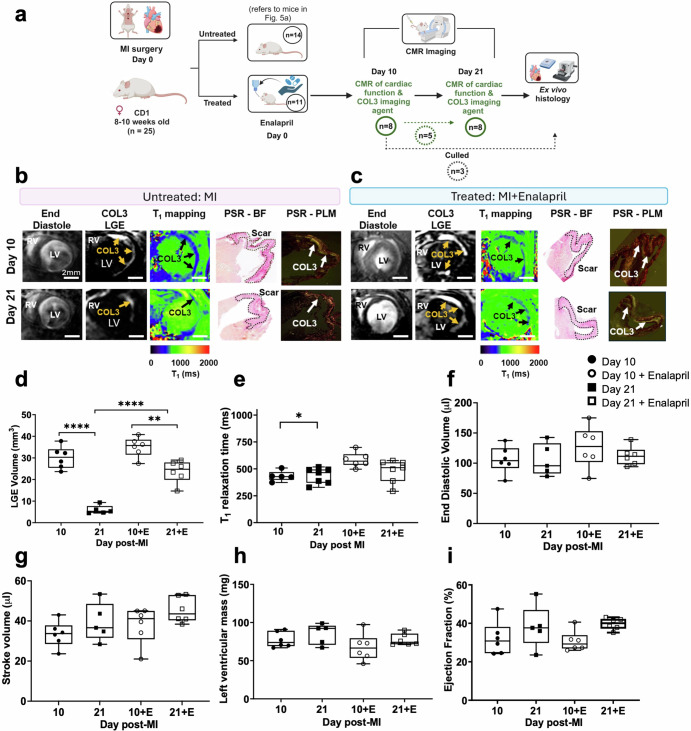
Molecular imaging of COL3 detects changes after enalapril treatment. **a** Experimental design. **b**, **c** At day 10, enalapril-treated mice show similar MRI signal enhancement and T_1_ relaxation times compared to untreated mice. At day 21, enalapril-treated mice show a significantly higher signal enhancement and reduced T_1_ relaxation values (blue colour) compared with untreated. **d**, **e** Quantification of LGE volume shows significantly higher COL3 remodelling in enalapril-treated mice compared with untreated mice at day 21. Quantification of gadolinium uptake showed higher T_1_ (lower gadolinium concentration) at day 21 compared with day 10 in untreated animals but similar values in enalapril-treated mice. **f**–**i** Cine MRI shows no significant changes in cardiac function between groups. LGE late gadolinium enhancement, and *P = 0.0233, **P = 0.0018, and ****P < 0.0001. RV right ventricle, LV left ventricle, PSR Picrosirius Red, BF bright field, PLM polarised light microscopy. Figure 6a was created with BioRender.com.

Next, we carried out an in vivo study to assess whether the [Gd-DOTAMA]_4_-CBP1 probe could detect changes in COL3 remodelling in mice treated with enalapril, a clinically used ACE inhibitor (Fig. 6a). We observed that enalapril-treated mice showed similar signal enhancement and probe uptake (low T_1_ values) at day 10 compared to untreated mice (Fig. 6b, c, d, e). Conversely, enalapril-treated mice showed a significantly higher volume of signal enhancement compared with untreated mice at day 21. However, the measured T_1_ value was not statistically significant (*P* = 0.1346). This data suggest that enalapril changes the kinetics of the natural turnover of COL3 post-MI and prolongs the presence of COL3 within the infarct. Such changes could be detected in vivo using the new probe. The imaging data were validated by histology using Picrosirius red staining (Fig. 6b,c). Functional CMR showed that mice with MI had dilated hearts and reduced ejection fraction in both groups (Fig. 6f–i). But despite the changes in COL3 remodelling observed with molecular imaging, no significant differences in cardiac geometry and function were observed between groups (Fig. 6f–i) suggesting that molecular changes in COL3 may precede structural or functional changes in the heart. Finally, imaging of the left ventricle from apex to base after injection of the COL3 probe enabled mapping of the spatial distribution of COL3 after MI (Supplementary Fig. 6). Interestingly, the distribution of COL3 within the infarct appears heterogeneous with a higher signal enhancement observed at the apex that decreases towards the base in both groups. Despite enalapril changing the time course of COL3 remodelling, it did not change the spatial distribution of COL3 within the infarct.

## Discussion

The lack of an imaging probe that specifically binds to COL3 and enables its in vivo imaging has hindered investigations into the potential diagnostic, therapeutic and functional roles of COL3 in myocardial fibrosis. The aim of this study was to develop and validate a gadolinium-based MRI molecular probe suitable for imaging COL3 in cardiac fibrosis after myocardial infarction. To achieve this goal, we first identified two small peptide sequences (CBP1 and CBP2)^41–43^ that were used to design candidate COL3 probes. Using in vitro screening studies, we then identified the probe that bound to COL3 with high affinity and specificity and used it to further detect COL3 remodelling after myocardial infarction non-invasively. Using this probe, we were able to detect spatiotemporal changes in COL3 turnover after myocardial infarction in vivo using molecular CMR in a murine model for the first time. We further demonstrated the sensitivity of the probe in detecting changes in COL3 remodelling following enalapril treatment, a clinically used ACE inhibitor.

When designing molecular imaging probes, it is important to identify and screen candidate peptides and prove that they are suitable for their intended target. Peptides are easy to synthesise, they bind to different targets (including proteins) with strong affinity (nM-µM) and their specificity can be proven via the synthesis of scrambled peptides^49^. Several methods have been developed for obtaining specific peptides including phage display^49^, one-bead-one-compound (OBOC) libraries^50^ and protein-protein interaction studies^21^.

Using candidate peptides, we synthesised monomeric COL3 imaging probes by conjugating a single DOTAMA chelator to their N-terminus. Because the chelator can be labelled with Eu(III) for in vitro binding experiments by TRF; with ^68^Ga(III) for in vivo biodistribution by PET; and with Gd(III) for MRI, these probes only differ in the metal ion (all trivalent) thus allowing consistent comparison between the Eu(III) TRF binding assays, biodistribution by PET, and MRI studies. Furthermore, the high thermodynamic and kinetic stability of Gd-DOTAMA complexes eliminates the risk of toxicity due to Gd(III) release^50–54^. In vitro binding assays by Eu(III) TRF yielded dissociation constants for COL3 binding in the low micromolar range, similarly to those reported for other MR molecular imaging agents targeting collagen or other ECM proteins^19,55,56^. However, the Eu-DOTAMA-CBP1 probe was superior to the CBP2 in terms of its specificity towards COL3. We also demonstrate that the affinity of CBP1 towards COL3 was not affected by the presence of the bulky, yet flexible, tetrameric moiety. This indicates that chemical modifications at the N-terminus of the CBP1 are well-tolerated.

Our data and those of others demonstrate the importance of not only using scrambled peptides but also using other ECM proteins to prove the specificity of a selected peptide towards the target of interest and to investigate any non-specific binding effects^19–22^. This is crucial to avoid any “off target effects” when the probes are administered in vivo. Accurate characterisation of the binding affinity and specificity of candidate probes require reliable binding assays. We have previously optimised the conditions to immobilise collagens into stable and reproducible gels and films with sufficient concentrations and also the conditions required for complete decomplexation of Eu(III) from the very stable Eu-DOTAMA complexes to activate Eu(III)^44^.

The CBP1 peptide sequence was derived from the N-terminus fragment of a platelet receptor that specifically binds to COL3^43^, which can reversibly inhibit COL3-mediated platelet aggregation by competing with the platelet receptor for COL3 binding sites. However, little is known about the details of the CBP1-COL3 binding interaction, including the location and number of COL3 binding sites, the binding topology and the type of non-covalent intermolecular bonds involved. Whether CBP1 binds on cross-linked versus un-crosslinked COL3 remains unknown. Despite the lack of details about the binding interaction, we have demonstrated that functionalisation at the N-terminus of CBP1 does not affect its binding towards COL3. Similarly to our study, the molecular interaction or binding site(s) of imaging probes to their target are frequently unknown. For example, the EP-3533 probe binds to COL1^19,28^ and EP-2104^20^ to fibrin (but not fibrinogen) but the details of the interaction with their targets are unknown. Yet, EP-2104R^57–59^ and a PET analogue of EP-3553^60^ have been trialled in humans for imaging thrombus and lung fibrosis, respectively.

The biodistribution profile of ^68^Ga-labelled monomeric probes obtained by in vivo PET/CT and ex vivo gamma-counting was favourable for both ^68^Ga-DOTAMA-CBP1 and ^68^Ga-DOTAMA-CBP2, exhibiting almost full clearance from the blood within 15–30 min with no significant unspecific uptake in any tissues. These observations agree with previously developed small peptide-based imaging agents of similar molecular weight and design^19,21,29,61^. The rapid elimination of the COL3 probes is beneficial for clinical translation because the post-contrast images can be acquired from 30 min onwards after probe injection. This time frame enables acquisition of baseline (pre-contrast) and (post-contrast) images in a single examination overcoming the need to wait a long time for target localisation and/or blood clearance that would necessitate separate pre- and post-contrast scans. Separate imaging sessions increase the hurdles for clinical translation because of difficulties in image co-registration, decreased patient compliance, difficulties in accessing the imaging unit and increased cost.

To validate in vivo the COL3 targeting capabilities of the CBP1 probes we first used a model of vascular remodelling that we previously showed to result in deposition of collagen and elastin within the remodelled aortic wall^47,62^. As compared to monomeric Gd-DOTAMA-CBP1, the [Gd-DOTAMA]_4_-CBP1 tetrameric probe demonstrated a more confined uptake and persistent signal within the remodelled aortic wall that co-localised with COL3 fibres seen on histology. The different kinetics of probe accumulation in COL3-rich regions between the monomer (MW = 1646 Da) and the tetramer (MW = 4460 Da) makes the latter more suitable for the intended applications, as it allows a better delineation of the remodelled aortic wall and, importantly, offers a longer image acquisition window after administration of the probe. A similar effect was observed when tropoelastin-targeting probes were developed^21,46^. In addition, it is worth noting that other peptide-based MRI probes targeting the ECM proteins also carry multiple Gd-chelates for optimal signal accumulation on the target (e.g. EP-3533/CM 101 targeting COL1^19,28^, EP-2104 targeting fibrin^20^).

On these grounds, the tetrameric [Gd-DOTAMA]_4_-CBP1 was chosen for COL3 imaging after MI in a well-established murine model of permanent occlusion of the proximal left anterior descending (LAD) coronary artery. The healing process post-MI is divided into three successive and overlapping phases: inflammation, proliferation, and maturation. Initially, immune cells respond to injury, followed by fibroblast activation and ECM protein deposition leading to the formation of scars rich in COL1, COL3 and elastin^63^. Although these phases have been described in different species and humans, the time scales and the location of the infarct itself varies. In contrast to humans, mice have a distinct septal coronary artery arising either from a separate ostium from the right sinus of Valsalva or as a branch of the right coronary artery^64,65^. Consequently, ligation of the LAD at the site of its emergence from under the left atrium in mice results in large MI involving the anterolateral, posterior, and apical regions of the heart. However, the septum is spared from infarction in mice as seen in our and other studies^35,39,66–68^. Thus, the distinct mouse coronary artery anatomy results in a different location of the infarct itself compared with humans and large laboratory animals.

We demonstrate that the tetrameric [Gd-DOTAMA]_4_-CBP1 probe enabled selective profiling of the natural turnover of COL3 after MI with the signal increasing at day 10, when COL3 is elevated, and decreasing at day 21 when COL3 is replaced by COL1. MRI signal enhancement co-localised with COL3 fibres as seen by histology. No enhancement was observed using the control scrambled probe or the clinically used non-targeted agent Gadovist. Importantly, in vivo monitoring of cardiac COL3 remodelling was detectable and feasible using a clinical scanner and clinically relevant field strengths, highlighting the translational potential of our work. In our study, the earliest short-axis inversion recovery images to visualise enhancement could be acquired about 25 min after injection of the probes. This timeframe does not affect visualisation of the uptake of the COL3 probe that binds to the target and stays within the infarcted myocardium significantly longer. However, this timeframe may explain the lack of enhancement after administration of Gadovist that washes out of the myocardium significantly faster. Previous studies that showed Gadovist enhancement of the scar obtained LGE images within 15 min post-injection of the agent^69^. Our findings using the COL3 probe agree with previous reports on the temporal changes in COL3 and COL1 after MI using ex vivo histology and biochemical analysis^70–73^. In rats, COL3 in the infarcted myocardium showed a transient 2.2-fold increase at 2 weeks post-MI but returned to baseline levels by 4 weeks^72^. Conversely, COL1 marginally increased in the first 2 weeks post-MI but increased by 2.2-fold from 4 to 10 weeks after MI. Functionally, the replacement of COL3 by COL1 was shown to increase cardiac stiffness and cause systolic dysfunction^70,73^. Similar changes in the ratio of COL3 to COL1 have been reported in patients with HF caused by ischaemic heart disease^74,75^. In pressure overload cardiac hypertrophy, increased COL1 content was associated with maladaptive remodelling whereas increased COL3 content was associated with adaptive remodelling^76^. Correction of increased pressure load in mice was characterised by an early increase of COL1 coinciding with reduced cardiac function followed by an increase in COL3 corresponding to improved cardiac function^77^. Such changes in collagen subtypes may be important determinants of cardiac function since different collagens have different biophysiological properties^4^. Thus, a COL3-binding probe may provide a tool to investigate the role of COL3 in cardiac disease. In conjugation with the existing COL1-binding probe, it can be used to investigate how changes in COL3 and COL1 may affect the biomechanical properties of the heart and its function.

We also tested whether the [Gd-DOTAMA]_4_-CBP1 probe could detect changes in COL3 remodelling after treatment. Using the ACE inhibitor, enalapril, we observed that although treated and untreated mice had similar COL3 remodelling at day 10 after MI, treated animals had significantly higher COL3 remodelling at day 21. This finding suggests that enalapril may prolong the presence of COL3 in the infarcted myocardium and this change can be detected non-invasively using the new COL3 MRI probe. ACE inhibitors decrease the formation of the vasoconstrictor angiotensin II and increase the formation of the vasodilator bradykinin^78^. Their main physiologic effects are vasodilation, natriuresis, enhancement of parasympathetic activity, and blunting of adverse remodelling, which are favourable in the setting of hypertension, heart failure, and MI^79–81^. Although the mechanisms/pathways by which ACE inhibitors affect myocardial fibrosis are complex, angiotensin II was shown to stimulate fibroblast proliferation and ECM synthesis through the TGF-β pathway^82–84^— a key regulator of collagen synthesis. Moreover, in patients with aortic aneurysms, another cardiovascular disease where fibrosis is crucial, treatment with ACE inhibitors increased the levels of circulating type III procollagen peptide (PIIINP), suggesting active synthesis of COL3^85^. The ability to non-invasively image COL3 directly may provide a tool to investigate the effects of ACE inhibitors in cardiac fibrosis and other cardiovascular diseases.

Our study has limitations. Firstly, we used a model of ischaemic cardiomyopathy by permanently occluding the LAD coronary artery that does not recapitulate other cardiac pathologies such as aortic stenosis leading to cardiac hypertrophy and fibrosis. Future studies in other animal models are needed to investigate the role of COL3 in cardiac diseases. Secondly, we assessed the effect of ACE inhibition on cardiac function only out to 21 days post-treatment. This limited time may not have been sufficient to elucidate the potential longer-term impact of ACE inhibition on cardiac function. Finally, we have not deciphered the binding sites, types of bonds and whether the COL3 probe binds to cross-linked or uncross-linked COL3. Future protein-protein interaction studies and work using drugs that are known to modulate crosslinking, such as lysyl oxidase inhibitors, could address these questions and investigate if collagen cross-linking affects binding of the COL3 probe.

Although COL3 is one of the major collagens involved in cardiac fibrosis-related diseases, in vivo detection of COL3 turnover has been hampered by the lack of a clinically feasible, non-invasive diagnostic tool. An imaging strategy using the COL3-specific MR contrast agent offers several potential advantages. Firstly, as the probe only binds to COL3 it allows for specific detection of areas of active COL3 remodelling in cardiac fibrosis. Secondly, COL3 MRI enables the direct localisation and quantification of the effects of treatments that aim to modulate collagen turnover. Thirdly, the adaptation of imaging sequences frequently used in human CMR and T_1_ mapping for small animal CMR at a clinical field strength makes our approach clinically translatable. Finally, the versatility of the probe to be chelated with radioisotopes makes it also suitable for nuclear/multimodal imaging, further increasing its translational potential.

In conclusion, we developed the first imaging probe specific to COL3 suitable for in vivo molecular imaging. The probe demonstrated strong affinity against COL3 and favourable properties for in vivo MR imaging. The probe uncovered previously undetectable changes in COL3 remodelling post-MI and allowed monitoring of therapeutic response to an ACE inhibitor. This probe may provide a non-invasive tool to investigate the unexplored roles of COL3 in cardiac fibrosis.

## Methods

### Peptide selection

The two peptides, DARKSEVQK (Collagen Binding Probe 1, CBP1) and TEFPLRMRDWLKN (CBP2), were identified from reported protein-protein interaction studies between collagen and other proteins^41,43^. The DARKSEVQK peptide is part of a platelet receptor, and it was shown to inhibit COL3-induced platelet aggregation and to have a higher binding affinity towards COL3 than COL1^43,86^. The TEFPLRMRDWLKN peptide is part of the secreted protein acidic and rich cysteine (SPARC) that binds to fibrillary collagens. The binding interaction between SPARC and collagens regulates collagen fibrillogenesis and influences the organisation and structure of collagen fibres in the ECM^42^.

### Peptide characterisation using expert protein analysis system (ExPASy)

Prior to in vitro and in vivo experiments, we used PEPTIDEMASS (ExPASy, SIB Bioinformatics Resource Portal) to check whether any enzymes could potentially cleave the candidate collagen-binding peptides. The enzymes identified in the database, included the Aps-N-endopeptidase, pepsin, and 2 proteinase K that are found in the digestive system and but not in blood. Therefore, we anticipated that the peptide-based probes will be delivered to the tissues intact. We also performed a sequence alignment using Protein Blast (National Institutes of Health, NIH) that revealed that both amino acid sequences used (CBP1 and CBP2) were not found in any other proteins apart from the platelet receptor and SPARC. This reduces the possibility that the peptides would form unspecific interactions by binding to other proteins.

### Synthesis of the MRI molecular probes

The monomeric molecular probes ending with a N-terminus free DOTAMA chelator and with a C-terminus free carboxylic group were custom synthesised by Peptide Synthetics Ltd (Hampshire, United Kingdom) with a purity of >95%. The DOTAMA collagen-binding peptides were complexed in house with Gd(III) to obtain a contrast agent for MR molecular imaging, or with Eu(III) for in vitro binding studies using Time-Resolved Fluorescence (TRF) plate assays^44^, or ^68^Ga(III) for PET biodistribution studies. Metal complexation of the DOTAMA derivatives was performed in-house, by reacting the desired lanthanide ion (Gd^3+^, Eu^3+^) in a 1.05:1 L:M molar ratio at pH 5.5 and room temperature. Reaction evolution was confirmed by xylenol orange method. Upon reaction competition, the pH was adjusted to 7 and the samples were lyophilised and stored at −20 °C. The final concentration of the Gd(III) and Eu(III) complex solutions was assessed by ICP-OES and/or BMS. For tissue co-localisation experiments, the CBP1 was also synthesised in the fluorescently labelled form by conjugating a Rhodamine B derivative to its N-terminus (compound Rh-CBP1).

To obtain the tetrameric probes, the collagen-binding peptide sequences were elongated with a N-acetylcysteine residue at the N-terminus (Ac-C-DARKSEVQK-COOH and Ac-C-TEFPLRMRDWLNK-COOH). The synthesis of [Gd-DOTAMA]_4_-CBPs (and their scrambled counterparts) was accomplished by adapting a modular approach based on the thiol/maleimide conjugation chemistry^46^. The cysteine-free thiol group was then used for conjugation with a preformed tri-lysine scaffold bearing four Gd-DOTAMA units using the well-established thiol/maleimide chemistry^46,87^, leading to compounds [Gd-DOTAMA]_4_-CBP1 and [Gd-DOTAMA]_4_-CBP2.

This approach relies on: (i) a heterobifunctional reagent containing a preformed tetrameric gadolinium-based imaging probe and a reactive maleimide group (compound [Gd-DOTAMA]_4_-MI, Supplementary Fig. 1a); (ii) a collagen binding peptide sequence elongated at the N-terminus with a cysteine residue (i.e. Ac-C-DARKSEVQK and Ac-C-TEFPLRMRDWLKN along with their scrambled versions, Ac-C-RDKKVAEQS and Ac-C-EWFMKDRLLNRPT, respectively). The N-terminus cysteine added to the collagen binding sequences provides the free thiol group needed for conjugation with the tetrameric Gd(III)-based unit through the maleimide/thiol chemistry (Supplementary Fig. 1b). Such a fast and clean conjugation reaction can be carried out in water solution with reagent molar ratio 1:1.

Compound [Gd-DOTAMA]_4_-MI was synthesised as described by Capuana et al. ^46^ and obtained with a purity >90%. The Ac-C-DARKSEVQK and Ac-C-TEFPLRMRDWLKN peptides (and their scrambled versions) were custom synthesised by standard Solid Phase Peptide Synthesis (SPPS) methods by Peptide Synthetics Ltd (Hampshire, United Kingdom), with a purity >95%. The thiol/maleimide coupling reaction was carried out under argon atmosphere by adding a solution of the cysteine containing peptide (approx. 100 mM, water solution, pH 6.7) to an equimolar amount of [Gd-DOTAMA]_4_-MI freshly dissolved in water (pH 6.7). The mixture was stirred at room temperature for 20 min. A small aliquot of the reaction mixture was analysed by UPLC to check for undesired excess of either the peptide or the [Gd-DOTAMA]_4_-MI reactant. If needed, a calibrated amount of the reagent was added to reach the proper reactant ratio. The product was purified using a GE AKTA Purifier 10 FPLC System, equipped with a Waters XTerra Prep C18 column (5 μm, 19 × 100 mm, cv 28.35 mL). The fractions containing the product were collected, and ACN evaporated under reduced pressure. The pH was adjusted to neutrality, and the solution was finally lyophilised.

The purity of the final products was assessed by analytical UPLC using an ACQUITY UPLC system equipped with a Peptide BEH C18 column (1.7 μm, 2.1 × 100 mm), applying a gradient elution (solvent A: 0.05% TFA in H_2_O; solvent B: CH_3_CN). The purity of the compounds was evaluated by the chromatographic peak area with UV-vis detection at λ = 220 nm.

[Gd-DOTAMA]_4_-CBP1 (MW 4460.32). Gadolinium content: 109 μg_Gd_/mg_powder_. UPLC purity: 90%. ESI + MS m/z calcd. for C_165_H_273_Gd_4_N_45_O_57_S: [M + 7H]^7+^ 637.94 (obsd.), 638.19 (calcd); [M + 6H]^6+^ 744.46 (obsd.), 744.39 (calcd); [M + 5H]^5+^ 892.49 (obsd.), 893.06 (calcd); [M + 4H]^4+^ 1115.60 (obsd.), 1116.08 (calcd).

[Gd-DOTAMA]_4_-SccCBP1 (MW 4460.32). Gadolinium content: 134 μg_Gd_ /mg_powder_. UPLC purity: 92%. ESI + MS m/z calcd. for C_165_H_273_Gd_4_N_45_O_57_S: [M + 6H]^6+^ 744.14 (obsd.), 744.39 (calcd); [M + 5H]^5+^ 893.05 (obsd.), 893.06 (calcd); [M + 4H]^4+^ 1116.00 (obsd.), 1116.08 (calcd).

[Gd-DOTAMA]_4_-CBP2 (MW 5106.14). Gadolinium content: 118 μg_Gd_ /mg_powder_. UPLC purity: 97%. ESI + MS m/z calcd. for C_199_H_316_Gd_4_N_52_O_61_S_2_: [M + 7H]^7+^ 729.69 (obsd.), 730.45 (calcd); [M + 6H]^6+^ 852.01 (obsd.), 852.02 (calcd). [M + 5H]^5+^ 1021.85 (obsd.), 1022.23 (calcd.).

[Gd-DOTAMA]_4_-SccCBP2 (MW 5106.14). Gadolinium content: 105 μg_Gd_ /mg_powder_. UPLC purity: 90%. ESI + MS m/z calcd. for C_199_H_316_Gd_4_N_52_O_61_S_2_): [M + 7H]^7+^ 730.22 (obsd.), 730.31 (calcd); [M + 6H]^6+^ 851.30 (obsd.), 851.86 (calcd); [M + 5H]^5+^ 1021.49 (obsd.), 1022.03 (calcd.)

### Europium(III) time resolved fluorescence (TRF) binding assays

The protocol to assess the binding affinity of the Eu(III)-labelled collagen probes to COL3 has been optimised previously^44^. Such an assay is based on the Eu(III) TRF which detects the amount of Eu(III)-labelled probes that bind to COL3 immobilised on a multi-well plate. The assay was used to measure the COL3 binding affinity of the monomeric Eu(III)-labelled probes (expressed as the dissociation constant, *K*_*d*_). The same assay was carried out with COL1, to estimate the selectivity for COL3 binding in respect to COL1. Potential unspecific binding effects associated with the assay were investigated by using two types of control experiments: (i) the scrambled versions of the Eu(III)-labelled probes were used as negative controls in TRF plate assays with immobilised collagen (either COL3 or COL1); and (ii) the collagen specific probes were assayed against elastin and bovine serum albumin (BSA) as control non-binding proteins. Briefly, increasing concentrations of the peptide (0.1–15 µM; 70 µL per well) were incubated in 96-well microplates pre-coated with the desired protein. Triplicate readings were taken per concentration and three independent experiments (plates) were used. After incubation of the peptide with the protein for one hour, the plates were washed with PBS to remove any free (unbound) peptide. To extract the Eu(III) from the probe, the wells were first treated with an acid solution for 90 min at 37 °C before adding a neutralising solution and the DELFIA^TM^ enhancement solution (30 min at room temperature). The plates were then read using a luminescence plate reader. These assays were used to determine the K_d_ values and fractional occupancies for the probes against COL3 and COL1, elastin and albumin as negative controls.

The binding interactions of the tetrameric Gd(III)-labelled probes with COL3 were studied by a competition assay, as Gd(III) is not suitable for TRF based assays. In competition assays, the binding of the Gd(III)-labelled tetrameric probe of interest to the target protein was measured by competition with the corresponding Eu(III)-labelled monomeric probe. Specifically, a row of wells was coated with collagen (COL3) and added with a constant amount of the Eu(III)-labelled probe of interest (e.g. Eu-DOTAMA-CBP1). Wells were then added and incubated with increasing concentrations of the corresponding tetrameric Gd(III)-labelled probes (e.g. [Gd-DOTAMA]_4_-CBP1). Displacement curves were then obtained by plotting the residual Eu(III) TRF against the concentration of the Gd(III)-labelled probe. Briefly, eight solutions were made containing a fixed concentration of Eu(III)-monomeric probe (5 µM) and increasing concentrations of the Gd-tetrameric probe (0.01–200 µM). Plates pre-coated with COL3 were simultaneously incubated with both compounds for 1 h at room temperature (triplicate wells per plate and 3 independent plates). After the incubation, the Eu(III) TRF assay protocol including the acid extraction and enhancement solution steps were used to measure the binding of the monomeric probe in the presence of the competing tetrameric probe, as previously described^44^. Displacement curves were then obtained by plotting the residual Eu(III) TRF against the concentration of the Gd(III)-labelled probe. Competition assays were carried out for both the CBP1 and CBP2 based probes.

### ^1^H-Nuclear magnetic relaxation dispersion (NMRD) measurements of the imaging probes in solution

To characterise the magnetic properties of the Gd-labelled collagen-binding candidate probes ^1^H-NMRD curves that represent the magnetic field dependency of the proton relaxivity (*r*_*1*_) were used. The ^1^H-NMRD profiles of Gd-DOTAMA-CBP1, [Gd-DOTAMA]_4_-CBP1, Gd-DOTAMA-CBP2 (1 mM solutions) were measured in both PBS and in 0.6 mM human serum albumin (HSA; Sigma Aldrich) at 25 and 37 °C in the frequency range 0.01–80 MHz. Longitudinal relaxation rates were recorded on a Stelar SMARtracer Fast Field Cycling NMR relaxometer (0.01–10 MHz) and a Bruker WP80 NMR electromagnet adapted to variable field measurements (20–80 MHz) and controlled by the SMARtracer PC-NMR console. The temperature was controlled by a VTC91 temperature control unit and maintained by gas flow. The temperature was determined according to previous calibration with a Pt resistance temperature probe. Relaxivity at 400 MHz was measured on a Bruker Avance 400 MHz spectrometer. Gd(III) concentration was confirmed by Bulk Magnetic Susceptibility (BMS) measurements (600 MHz, Bruker Avance Spectrometer, 5 mm BBFO probe in D_2_O) and/or ICP-OES.

### Radiolabelling the probes with Gallium-68 for PET/CT in vivo and ex vivo biodistribution

Gallium-68 (^68^Ga) was eluted from the generators onsite at the time of the experiments. Radiolabelling of the respective DOTAMA-CBP compound was performed as follows: DOTAMA-CBPs were dissolved in water (Baxter water for irrigation) to a final concentration 4 mg/mL. In an Eppendorf, 25 µL of the DOTAMA-CBP solution was added. Sodium acetate (30%NaOAc, 20 µL, pH 5) buffer was added to this solution followed by the addition of 200 µL ^68^Ga (~100 MBq). The solution was then heated to 50 °C for 30 min. After the reaction was removed from heat, the pH was adjusted to pH 7 by adding sodium bicarbonate (1 M, 10 µL). Labelling efficiency was followed by radio thin-layer chromatography (TLC) and HPLC. Radio TLCs were performed using silica gel IBF-2 Baker plates, 10% NH4OAc 50:50 in MeOH with 5 µL of the sample added. For the HPLC, a C18 column was used (MeCN + 0.1% TFA and H_2_O + 0.1% TFA, gradient) with 10 µL of the sample measured.

### Animal studies

All animal procedures were performed in accordance with the guidelines of the United Kingdom Home Office Animal (scientific procedures) Act 1986, project license number PP8261525. Ethical approval was granted by King’s College London’s Animal Welfare and Ethical Review Body.

### Biodistribution of ^68^Ga-DOTAMA-CBP1 and ^68^Ga-DOTAMA-CBP2

In vivo biodistribution imaging was carried out using a positron emission tomography/computed tomography (PET/CT) scanner (NanoPET/CT scanner; Mediso, Hungary. Male apolipoprotein E knockout (ApoE^−/−^) were anesthetised with 4% isoflurane mixed with 1% medical oxygen and maintained at 2% isoflurane for the PET/CT experiment. Mice were injected intravenously (i.v.) with either ^68^Ga-DOTAMA-CBP1 or ^68^Ga-DOTAMA-CBP2 (12MBq/mouse for both probes). To verify anatomical structures, CT images were obtained after the PET imaging with a 55 kVp voltage. PET images were acquired continuously up to 90 min post-injection with a 256 × 256 matrix size and a resolution of 1 mm/pixel. PET and CT images were reconstructed using the manufacturer software with a voxel size of 0.4 mm and analysed with a VivoQuant 2.5 software (Invicro, Boston, MA, USA). The data acquired with the CBP1 and CBP2 imaging probes were reconstructed in 15-minute bins up to 90 min. The PET/CT images were merged using the software’s automated fusion function. Additionally, the software was used for region-of-Interest (ROI) analysis and results were reported as %ID/g for each organ. Following the in vivo PET/CT imaging, the animals were euthanised and tissues extracted for ex vivo gamma counting (1282 CompuGamma gamma counting, LKB Wallac, Finland).

### Murine model of vascular remodelling

Male ApoE^−/−^ mice were bred in-house and underwent surgery at 8–10 weeks of age (*n* = 6). Mice were anesthetised with 4% isoflurane mixed with 1% medical oxygen and anaesthesia was maintained at 2% during the surgery as previously reported^47^. Briefly, mice underwent a laparotomy to expose the abdominal aorta, renal and iliac arteries. Two ligatures were inserted around the aorta (surgical silk, size 4–0, Aragó, Zaragoza, Spain), the first below the renal arteries and the second above the iliac bifurcation. A 30 G needle was introduced into the abdominal aorta at the level of the iliac bifurcation, via an aortic puncture. 1 mL of saline was infused into the aorta in three boluses. Finally, the needle was removed, and the puncture was repaired. The muscle and skin incisions were sutured (5-0 Vicryl, Ethicon Inc, Somerville, NJ), and the animals were allowed to recover. Mice were switched to a high-fat diet (HFD) containing 21% fat from lard and 0.15% (wt/wt) cholesterol (Special Diets Services, Witham, United Kingdom), on day 2 post-surgery for 12 weeks prior to the imaging experiments.

### Murine model of myocardial infarction

Female CD1 mice were purchased from Charles River Laboratories (Margate, United Kingdom) and underwent surgery at the age of 8–10 weeks old (*n* = 25). Mice were anesthetised with an intraperitoneal (i.p.) injection of Ketamine at 100 mg/ml & Medetomide hydrochloride at 1 mg/ml and analgesia was administered i.p. Mice were intubated and placed on a ventilator (tidal volume of 150 µL/stroke and ventilation rate to 140 strokes/min, MiniVent). A left thoracotomy in the 3rd intercostal space was performed to expose the heart. The left anterior descending (LAD) artery was ligated using an 8–0 ethilon suture, 1–2 mm below the tip of the left atrium, resulting in a proximal LAD occlusion. The chest cavity and the skin were closed with a 5–0 ethilon suture. An antidote (Antisedan, 5 mg/ml, inject 10 µL/g) was injected i.p. and the animals were allowed to recover. Mice were split into two groups: untreated (*n* = 14) or treated with enalapril (*n* = 11; 20 mg/kg/day) added in drinking water immediately after the surgery. In vivo cardiac MRI was performed at days 10 and 21 post-surgery.

### In vivo MRI

Imaging was performed using a 3 Tesla Philips Achieva MR scanner (Philips Healthcare, Best, The Netherlands) equipped with a clinical gradient system (30 mT/m, 200 mT/m per millisecond).

### Vessel wall imaging

Anaesthesia was induced with 4% and maintained with 1–2% isoflurane during the MRI experiments. Mice (*n* = 3 per group) were placed supine on a single-loop surface coil (diameter = 47 mm) and the abdominal aorta was imaged up to 2 h post-injection of the monomeric (i.v. 0.2 mmol/kg, 0.2 mmol/kg Gd), the next day with the corresponding tetrameric collagen-binding probe (i.v 0.2 mmol/kg, 0.8 mmol/kg Gd) and the 3rd day using the scrambled probes (*n* = 2–3 per probe; i.v. 0.2 mmol/kg). This timeline ensures complete washout of one probe from the tissue before the administration of another probe. Previous work using other targeted probes showed clearance from both the blood and vessel wall 24 h after the administration of a probe^21^.

Following a 3D GRE MRI scout scan with a FOV = 200 × 101 × 14 mm, acquisition matrix = 336 × 1693 mm, slice thickness = 1 mm, TR/TE = 12/6.0 ms, flip angle = 30°, TFE factor = 1, contrast enhanced MR angiography (MRA) images were acquired to visualise the vasculature extending from the left renal branch to the iliac bifurcation (where vascular injury was surgically-induced) with: FOV = 35 × 35 × 16 mm, acquisition matrix = 232 × 233 mm, slice thickness = 0.5 mm, TR/TE = 28/6.0 ms, flip angle = 40°, TFE factor = 1 and scan time = 3.34 min. The maximum intensity projection images were used to plan the subsequent scans. A 2D-Look-Locker sequence planned perpendicular to the vessel was used to calculate the inversion delay time to suppress the signal from blood was acquired with a FOV = 30 × 30 mm, acquisition matrix = 80 × 78 mm, slice thickness = 2 mm, TR/TE = 19/8.5 ms, flip angle = 10°, TFE factor = 1 and scan time = 0.53 min. Then, late gadolinium enhanced (LGE) MR images were obtained to visualise probe uptake in the tissue either as a result of unspecific retention or binding of the probe to its target. An inversion-recovery (IR) 3D fast gradient echo sequence was used with: FOV = 35 × 35 × 12 mm, acquisition matrix = 352 × 3490 mm, slice thickness = 1 mm, TR/TE = 27/8.2 ms, repetition time between subsequent inversion recovery pulses = 1000 ms, flip angle = 30°, TFE factor = 12 and scan time = 12 min. Serial IR images were acquired up to 2 h post-injection of the probe.

All MR imaging data was exported in a digital imaging and communication in medicine (DICOM) format and subsequent analysis was performed using the open-image analysis platform Horos 3.0 (Horos Project, Annapolis, MD, USA). Vascular remodelling was quantified slice-by-slice by manually segmenting the visually enhanced segment of the aorta seen on the images acquired along the aorta. DICOM images were loaded into the user workspace and LGE images were displayed alongside the transverse MRA images. The MRA images were then resampled so that the slice thicknesses of the scans matched that of the LGE. The images were then aligned and fused. The co-registered images were used to guide the segmentation of the LGE images which was performed using the closed polygon tool and when the entire vessel was segmented the regions of interest (ROI) containing areas of enhancement (mm^2^) were exported. The sum of the LGE areas was calculated and reported as the total area of enhancement (mm^2^) which was then converted into the volume of enhancement (mm^3^), by multiplying the total area with the slice thickness (1 mm).

### Cardiac MR imaging

Four ECG pads (similar to those used in patients) were cut to size prior to use. The adhesive gel of the pad was removed and replaced by Nuprep skin prep gel (Weaver and Company, USA) to improve the conductivity between the ECG pads and the mouse’s paws and help maintain a stable ECG recording during the scan. Each ECG pad was carefully placed over the dorsal side of the mouse’s paws and individually taped to the platform to ensure the pad did not displace or detach during the scan which would result in loss of the ECG reading. The ECG module was further stabilised using a sandbag.

Anaesthesia was induced with 4% isoflurane and maintained with 1–2% during the scans. Mice were placed in a prone position on a single-loop surface coil (diameter = 23 mm). At day 10 post-MI, the left ventricle was imaged for up to 1.5 h post-injection of the lead compound ([Gd-DOTAMA]_4_-CBP1, i.v. 0.2 mmol/kg, 0.8 mmol/kg Gd) (*n* = 7). Mice were then imaged on days 11 and 12 after injection of the scrambled probe and Gadovist, respectively (*n* = 2–3 per group; i.v. 0.2 mmol/kg; 24 h apart between scans). Another cohort of mice (*n* = 7) were imaged at days 21, 22 and 23 using analogous experiments. Mice treated with enalapril were imaged after injecting [Gd-DOTA]_4_-CBP1 on days 10 and 21 post-MI (*n* = 7 per group).

For CMR scans, coronal and transverse scout images were used to locate the heart. Following these scans, a two-chamber short-axis (SA) cardiac cine scan was planned. This was achieved by placing the slice in a mid-left ventricular (LV) position, perpendicular to the long-axis of the LV. The acquisition parameters were: FOV = 35 × 35 mm, acquisition matrix = 176 × 175 mm, slice thickness = 1 mm, TR/TE = 14/7.7 ms, flip angle = 20°, TFE factor = 1 and scan time = 0.38 min. Then, four-chamber images were acquired by placing a slice perpendicular to the SA images, running parallel to the connection points between the left and right ventricle. The slice was centred in the middle of the LV and in the coronal image the slice was aligned along the long-axis covering the apex through the base. The acquisition parameters were: FOV = 35 × 35 mm, acquisition matrix = 176 × 175 mm, slice thickness = 1 mm, TR/TE = 13/6.4 ms, flip angle = 20°, TFE factor = 1 and scan time = 0.40 min. To obtain short-axis two-chamber images, a single slice was positioned perpendicular through the LV in both the two and four-chamber views. The acquisition parameters were: FOV = 35 × 35 mm, acquisition matrix = 232 × 230 mm, slice thickness = 1 mm, TR/TE = 15/6.9 ms, flip angle = 40°, TFE factor = 1 and scan time = 1.55 min. Once the plane for the short-axis images was confirmed, a 2D cine short-axis scan covering the whole heart was acquired (typically 6–8 slices, 1 mm slices depending on the size of the heart). The acquisition parameters were: FOV = 35 × 35 mm, acquisition matrix = 176 × 177 mm, slice thickness = 1 mm, TR/TE = 13/6.5 ms, flip angle= 40°, TFE factor = 1 and scan time = 12.40 min.

The two-chamber cine short-axis scan was used to plan a single slice 2D-Look Locker (LL) sequence to calculate the inversion delay time to suppress the remote myocardium for LGE imaging. The 2D-LL was acquired with: FOV = 35 × 35 mm, acquisition matrix = 72 × 70 mm, slice thickness = 1 mm, TR/TE = 9/4.1 ms, flip angle = 6.4°, TFE factor = 1 and scan time = 2.2 min. Subsequently, LGE MR images to visualise probe uptake in the infarcted myocardium either as a result of unspecific retention or binding of the probe to its target were obtained using a short-axis inversion recovery (IR) 3D fast gradient echo sequence. Serial IR images were acquired starting at 30, 60 and 90 min post-injection of the probe to determine the optimal imaging time point resulting in the highest enhancement of the infarcted myocardium. Images were acquired with a FOV = 35 × 35 × 8 mm, acquisition matrix = 116 × 117 mm, slice thickness = 1 mm, TR/TE = 6.8/2.9 ms, repetition time between subsequent inversion recovery pulses = 1000 ms, flip angle = 40°, TFE factor = 3 and scan time = 9.5 min. Both 2D-LL and 3D-IR images were acquired by simulating the heart rate to 60 bpm (irrespective of the mouse’s heart rate). This approach was used to ensure that the inversion pulse was spaced every 1000 ms (one RR interval) for consistent recovery of the magnetisation and that within each RR interval there was sufficient time to wait for the inversion delay (about 250 ms) to supress the remote myocardium. This would not have been possible when using the recorded ECG in mice (typically 350–500 bpm) resulting in an RR interval of 133–171 ms. Finally, T_1_ mapping was performed ~1 h post-injection of the agent using a 2D gradient echo Look-Locker-based sequence that employs a non-selective inversion pulse with inversion times ranging from 1 ms to 10,000 ms, followed by thirty segmented readouts for thirty individual inversion recovery images. We maximised the number of readouts to sample the recovery of the magnetisation efficiently given the short RR interval duration in mice. For T_1_ mapping, the acquisition parameters were: FOV = 35 × 35 mm, acquisition matrix = 116 × 110 mm, slice thickness = 2 mm, TR/TE = 7.2/3.6 ms, flip angle = 15°, TFE factor = 5 and scan time = 4.2 min. Given the fast heart rates in mice, the longitudinal magnetisation does not have sufficient time to recover before the next inversion pulse is applied. We therefore implemented ‘blanking’ of the heartbeats, such that for every three heartbeats two were omitted resulting in an “effective RR interval” that was prolonged compared with the actual RR interval using the following equation:

$${Effective\; RR}=\frac{60,000}{{Heart\; rate}}\times[{number\; of\; blanked\; beats}+1]$$

Note: In this equation the [+1] is the fixed beat that we use to acquire the data in the sequence.

All MR imaging data was exported in a digital imaging and communication in medicine (DICOM) format and subsequent analysis was performed using the open-image analysis platform Horos 3.0 (Horos Project, Annapolis, MD, USA). K-space raw data was also exported, using the Gyro Tools application, for offline reconstruction of the T_1_ maps.

The 2D short-axis cine images covering the LV were analysed using the MRHeart plugin to calculate cardiac function. The end systolic (ES) and end diastolic (ED) phases were identified in each slice. The epicardial and endocardial contours where manually segmented at end systole and end diastole for every slice and used to calculate the end systolic volume (µL), end diastolic volume (µL), ejection fraction (%), stroke volume (µL) and left ventricular mass (mg).

Probe uptake within the infarcted myocardium was quantified on a slice-by-slice basis by manually segmenting the visually enhanced area seen on the LGE images. To aid segmentation of signal enhancement, the LGE images were co-registered and fused with the 2D short-axis cine images containing anatomical information. The segmented area of signal enhancement calculated per slice (mm^2^) was summed and multiplied by the slice thickness to calculate the total volume of enhancement per heart (mm^3^).

The T_1_ maps were reconstructed off-line (using SENSE6 and coil maps estimated by ESPIRiT7 for multi-channel rabbit scans) to reconstruct the intermediate T_1_ weighted images using an in-house developed MATLAB script. When reconstructing the data offline, we observed that the conventional T_1_ mapping fit was susceptible to flow artefacts and fluctuations in heart rate. To overcome this challenge, a combination between conventional and dictionary mapping was used as previously reported^88^. To generate the dictionary, the typical 2-parameter fit model was used based on the following equitation:

$${M}_{z}(t)={M}_{o}(1-2{e}^{\frac{-t}{T1}})$$

where, M_z_ = longitudinal relaxation, M_o_ = initial magnetisation, t = time

This model was then used to simulate the magnetisation for a range of T_1_ relaxation times (100:1:10,000 ms, minimum value: step size: maximum value) and generate a dictionary of signal evolutions. The inner product between the dictionary and the temporal evolution of the acquired T_1_-weighted images was evaluated pixel-wise to identify the best match in the dictionary and reveal the corresponding T_1_ value, as commonly used in magnetic resonance fingerprinting^89^. The obtained T_1_ maps were further analysed in Horos.

### Tissue collection and processing

After imaging, the abdominal aorta between the renal branches and the iliac bifurcation was collected, washed in saline, pinned down onto a cork, and fixed in 4% formaldehyde for 48 h at 4 °C. After imaging, hearts were collected, flushed with saline, and fixed in 4% formaldehyde for 48 h at 4 °C.

### Histology

Aortas and hearts were dehydrated, paraffin-embedded and transversely sectioned (5 μm for the aorta and 7 μm for the hearts). Aortas were stained with Picrosirius red (Abcam, Cambridge, United Kingdom) to assess the collagen. Hearts were stained with both Masson’s trichrome (Sigma–Aldrich, Dorset, United Kingdom) and Picrosirius red to assess cardiac morphology and collagen content, respectively. Immunohistochemistry (IHC) was used to detect COL1 and COL3 in the myocardium using an anti-mouse rabbit polyclonal antibody (ab21286; dilution 1:100 and ab7778; dilution 1:30, respectively). Briefly, slides were first deparaffinised and rehydrated before antigen retrieval using a citrate buffer (5 min, 99 °C twice). Slides were then washed in PBS three times for 5 min. Endogenous peroxidase was inhibited using 3% oxygenated water in methanol for 10 min. Slides were then washed in PBS before blocking with 10% donkey serum in 1% bovine serum albumin (BSA) in PBS for 1 h at room temperature. Sections were incubated in primary antibody (diluted in 2% donkey serum and 1% BSA in PBS, at the respective concentrations) overnight at 4 °C. The next day sections were tempered at room temperature for 30 min before washing in PBS. The sections were then incubated with the secondary antibody (donkey anti-rabbit IgG biotinylated; ab207999; 1:1000 dilution) for 1 h at room temperature. After the incubations, the sections were washed in PBS and incubated in ABC kit (Vectastain; PK6100) for 30 min. Following the incubation, the sections were washed in PBS and revealed using 3,3′-Diaminobenzidine (DAB) for 3 min per section and then washed in distilled water. Counter staining was complete using Gill’s haematoxylin solutions (Merck; GHS332) for 20 s. Sections were washed in running water and then immersed one time in 1% acid alcohol followed by dehydration and mounting.

### Image analysis

Histology images were digitised using a slide scanner (Hamamatsu NanoZoomer S360) and analysed using ImageJ. For IHC staining and Picrosirius red staining seen under polarised light, images were taken using a Leica DM2700 P Microscope. Cross-sectional cardiac images of the left ventricle were matched with histological sections utilising papillary muscles as landmarks seen on both the MRI and histology.

### Statistical analysis

GraphPad Prism 8.00 (GraphPad Software, Inc., La Jolla, California, USA) was used. Normality was assessed by Q-Q plots and the Shapiro-Wilk test. Equal variances were assessed with the Bartlet’s test. For normally distributed variables with equal variances, a one-way ANOVA test followed by a Tukey’s multiple comparison test (e.g., LGE volumes, R_1_ of infarct and remote myocardium) was performed. *P* < 0.05 was considered statistically significant (*P = 0.0233, **P = 0.0018, ****P < 0.0001). Data are presented as mean ± standard deviation (SD).

## Supplementary information

Supplementary material

## Data Availability

No datasets were generated or analysed during the current study.
