# Supplementary material for: Non-invasive in vivo imaging of changes in Collagen III turnover in myocardial fibrosis
Source: Npj Imaging. 2024 Sep 17;2:33. doi: 10.1038/s44303-024-00037-z (PMC11408249; doi:10.1038/s44303-024-00037-z)
Supplement: Supplementary file 1 — Supplementary material [file 44303_2024_37_MOESM1_ESM.pdf]

Supplementary material

Title: “Non-invasive in vivo imaging of changes in Collagen III turnover in myocardial fibrosis”

Authors: Nadia Chaher, Sara Lacerda*, Giuseppe Digilio*, Sergio Padovan, Ling Gao, Begoña Lavin, Rachele Stefania, Carlos Velasco, Gastão Cruz, Claudia Prieto, René M. Botnar, Alkystis Phinikaridou

*These authors contributed equally

Supplementary data – Figures

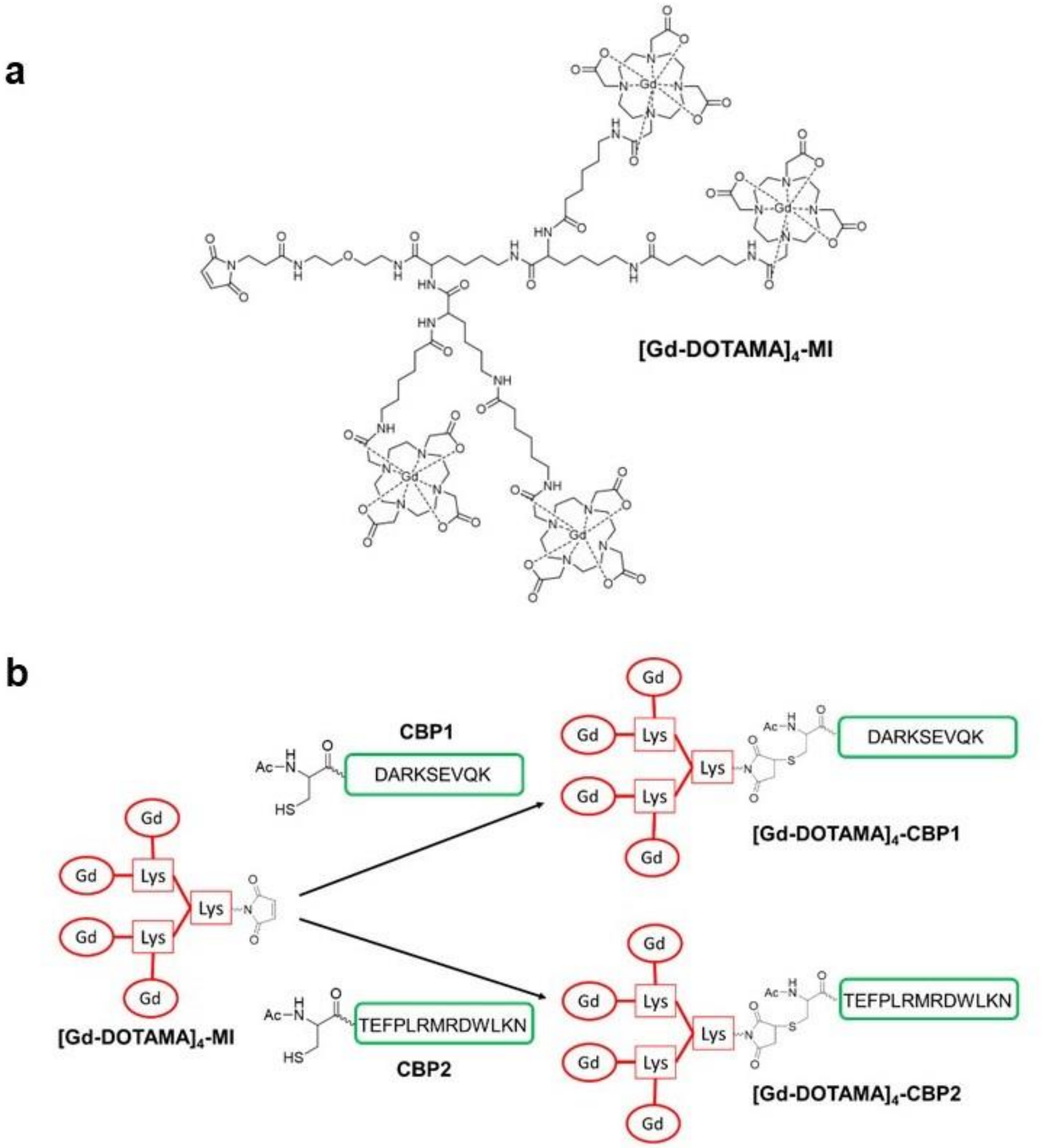

Supplementary Figure 1: Chemical structure and synthetic approach to produce the tetrameric probes. **a.** Chemical structure of the heterobifunctional reagent [Gd-DOTAMA]$_4$-MI. **b**. Synthetic approach to obtain the tetrameric, Gd(III)-based MRI probes targeting COL3.

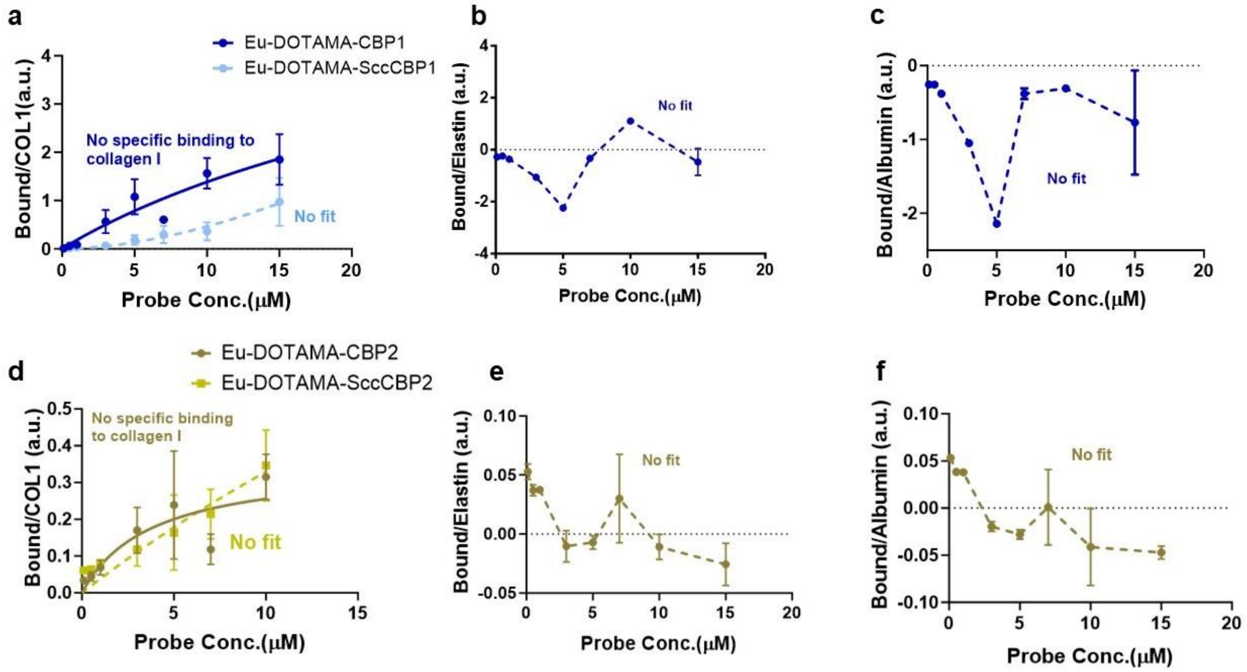

Supplementary Figure 2: **Binding assays of the Eu-DOTAMA-CBP1 and CBP2 probes against immobilised collagen 1 (COL1), elastin and albumin. a.** The Eu-DOTAMA-CBP1 shows no specific binding to COL1. **b,c**. The Eu-DOTAMA-CBP1 does not bind to elastin and albumin (no fit). **d.** The Eu-DOTAMA-CBP2 shows non-specific binding to COL1. **e,f**. The Eu-DOTAMA-CBP2 does not bind to elastin and albumin (no fit). CBP= collagen binding peptide; SccCBP= scrambled version of the collagen binding peptide; Eu= europium.

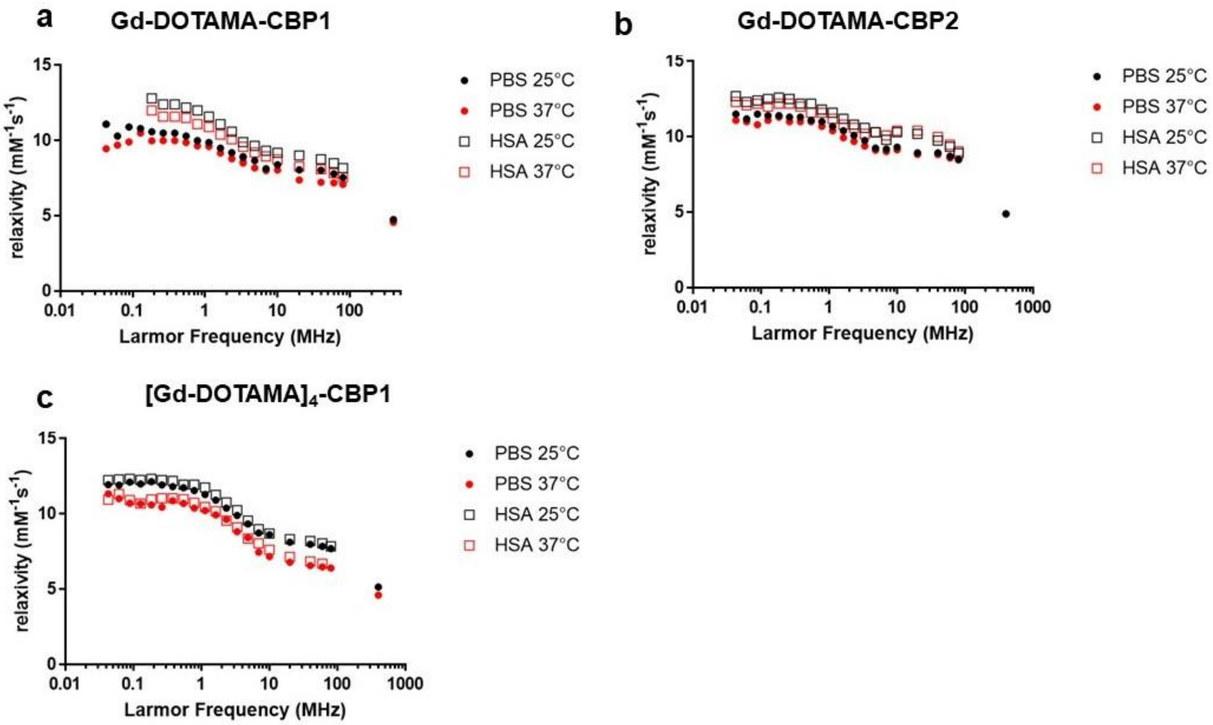

Supplementary Figure 3: ^{1}H nuclear magnetic relaxation dispersion profiles (^{1}H-NMRD) acquired in PBS and in 0.6mM human serum albumin (HSA), at 25 and 37°C. a. Gd-DOTAMA-CBP1. **b**. Gd-DOTAMA-CBP2. **c.** [Gd-DOTAMA]$_4$-CBP1.). CBP= collagen binding peptide; Gd= gadolinium; PBS= phosphate-buffered saline; HSA= human serum albumin.

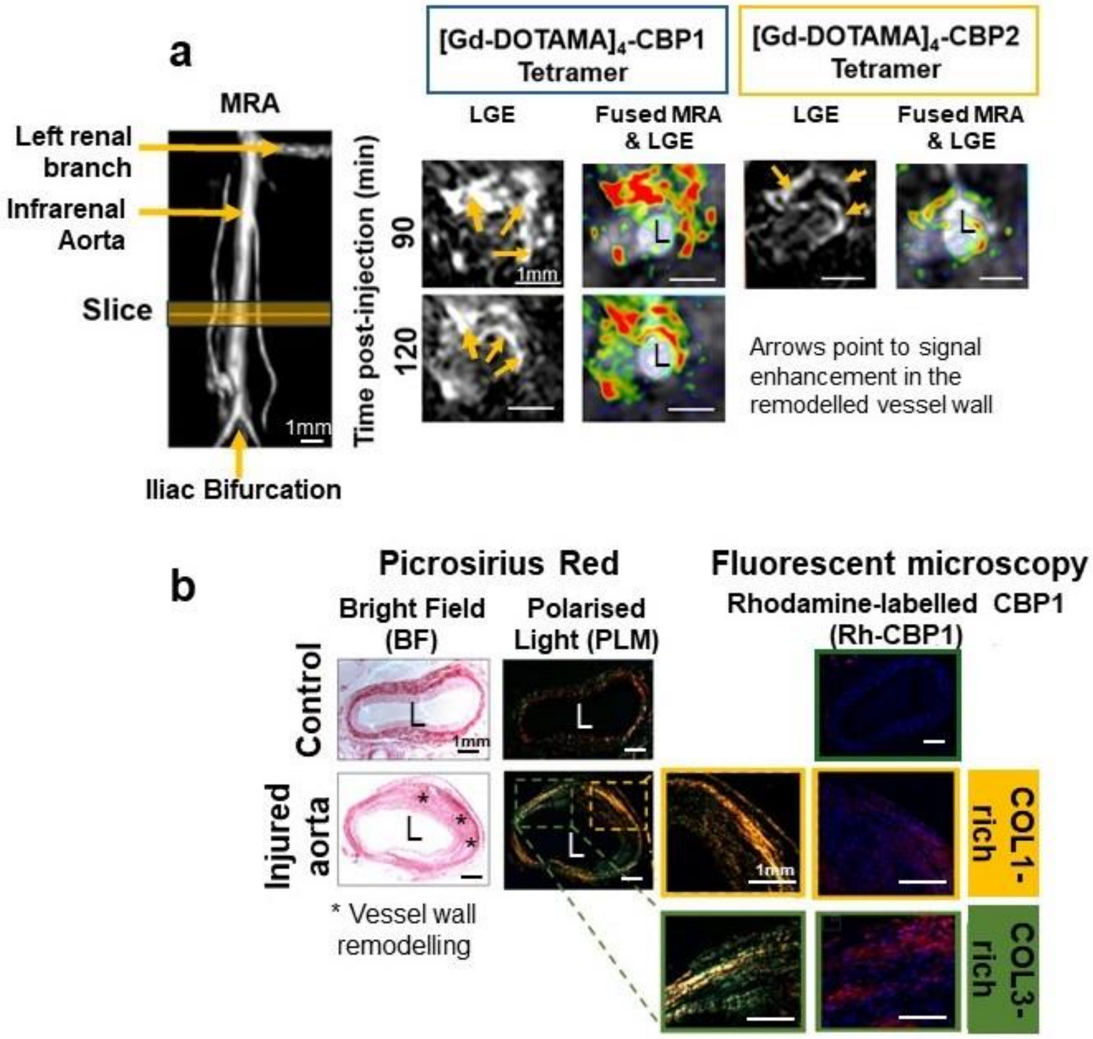

Supplementary Figure 4: In vivo vessel wall imaging using the tetrameric CBP1 and CBP2 imaging probes and ex vivo histology. a. [Gd-DOTAMA]$_4$-CBP1 shows stable enhancement up to 2 hours post-injection and stronger MRI signal compared with [Gd-DOTAMA]$_4$-CBP2. **b.** Picrosirius red stained sections under bright and polarised light show collagen remodelling in the injured aorta containing a mixture of COL1 (yellow) and COL3 (green) fibres. Microscopy of tissue sections using the fluorescently labelled CBP1 peptide demonstrates little uptake in the control aorta. However, the fluorescent signal from CBP1 co-localises with COL3 fibres but not with COL1 fibres within the remodelled vessel wall. MRA= magnetic resonance angiography; LGE= late gadolinium enhancement; Rh= rhodamine; PSR= Picrosirius Red; BF= bright field; PLM= polarised light microscopy.

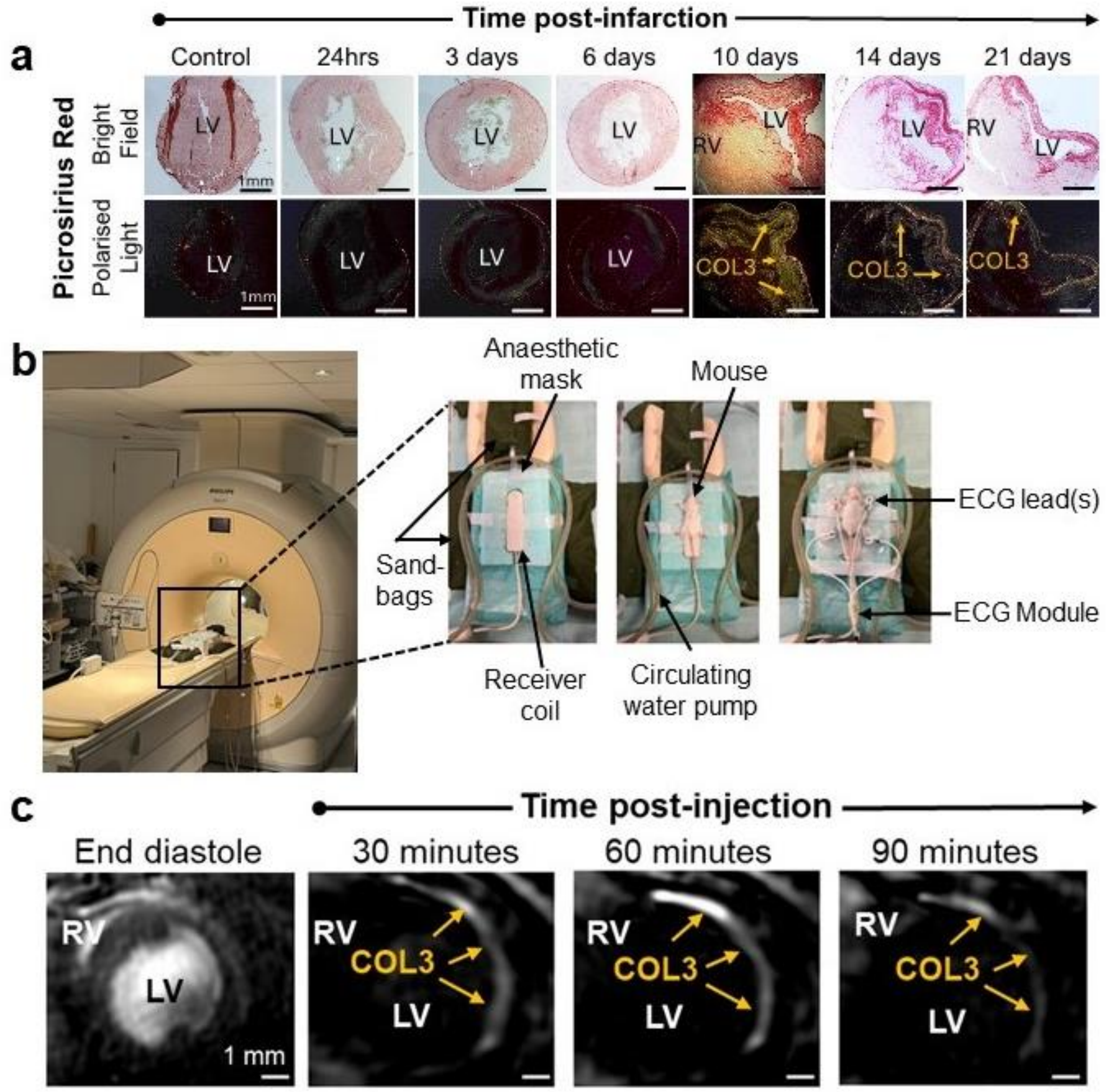

Supplementary Figure 5: Histological and in vivo cardiac MRI characterisation of collagen expression after MI. a. Based on Picrosirius red staining, collagen remodelling (red fibres) starts within the first week post-MI. COL3 (green under polarised light) is elevated at day 10 and by day 21 is replaced by COL1 (yellow/ orange). **b.** Setup for cardiac MRI in mice at a clinical 3 Tesla scanner. **c.** MRI of COL3 at different time points after injection of the $[Gd\text{-}DOTAMA]_4$-CBP1 probe post-MI at day 10. Uptake of the $[Gd\text{-}DOTAMA]_4$-CBP1 in the infarcted myocardium is observed at 30 minutes, peaks at 60 minutes and decreases by 90 minutes post-injection (n=3 mice). LV= left ventricle; RV= right ventricle; ECG= electrocardiogram.

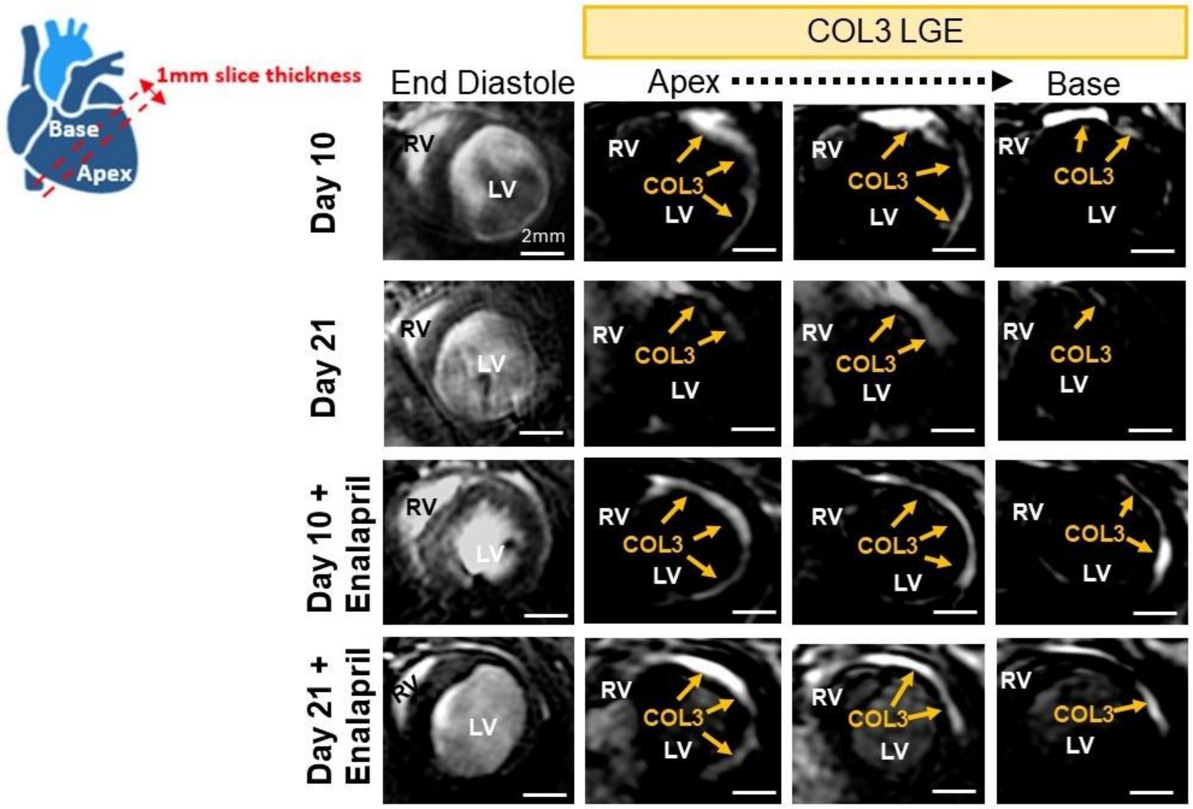

Supplementary Figure 6: Spatial distribution of the [Gd-DOTAMA]$_4$-CBP1 imaging probe in treated and untreated mice after MI. In untreated mice, COL3 remodelling seen as signal enhancement in the left ventricle is higher at the apex and decreases towards the base at day 10. At day 21, COL3 remodelling decreases but the majority of the COL3-enchacement still appears in the apex. In treated mice, the distribution of COL3 is similar to that observed in untreated mice with COL3 accumulating in the apex and decreasing towards the base at day 10. However, in treated mice COL3 remodelling increases with COL3 accumulating at the apex and extending towards the mid-heart, at day 21. LV= left ventricle; RV= right ventricle; LGE= late gadolinium enhancement. Schematic was created with BioRender.com
